# Supplementary material for: Genome-wide screen overexpressing mycobacteriophage Amelie genes identifies multiple inhibitors of mycobacterial growth
Source: G3 (Bethesda). 2024 Dec 5;15(2):jkae285. doi: 10.1093/g3journal/jkae285 (PMC11797047; doi:10.1093/g3journal/jkae285)
Supplement: jkae285_Supplementary_Data [file jkae285_supplementary_data.pdf]

Supplemental Figure 1: Shown are the results of representative cytotoxicity assays for the 76 Amelie genes screened in this study. Each strain was spotted in triplicate alongside *M. smegmatis*/pExtra-Fruitloop*52* (+) and pExtra-Fruitloop*52*I170S (-) control strain in 7H10 Kan supplemented with 0, 10, or 100 ng/ml aTc. In all experiments, 10^0 to 10^{-5} dilutions are shown. Plates were monitored over 4 or 5 days at 37℃, with results shown to best illustrate effects on colony color and size. Colony color was scored using the indicated key shown at the bottom of the data card.

Gene *1*; Score *0*

Images taken after 5 days at 37 ºC

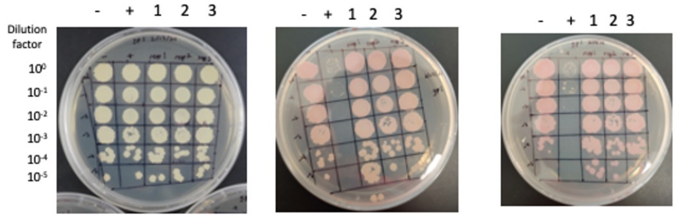

0 ng/ml aTc | 10 ng/ml aTc | 100 ng/ml aTc

Lane	Gene ID	Plasmid name	Gene name	Toxic/Non-toxic	Colony color on 100 ng/ml aTc plate*
- Non-toxic control	--	pExTra03	Fruitloop *52* mutant	Non-toxic	++
+ Toxic control	--	pExTra02	Fruitloop *52*	Toxic	-
1		pExTra-Amelie 01	Amelie 01 replicate 1	Non-toxic	++
2		pExTra-Amelie 01	Amelie 01 replicate 2	Non-toxic	++
3		pExTra-Amelie 01	Amelie 01 replicate 3	Non-toxic	++

*Key: NG (no growth) - (no pink color) +(faint pink color) ++(obvious pink color) +++ (dark pink color)

Gene *2*; Score *0*

Images taken after 5 days at 37ºC

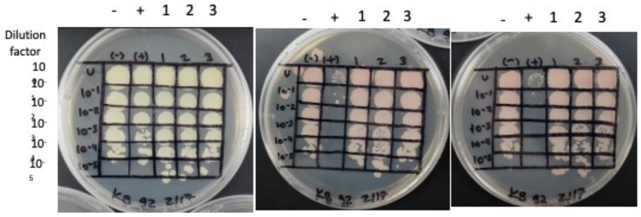

0 ng/ml aTc | 10 ng/ml aTc | 100 ng/ml aTc

Lane	Gene ID	Plasmid name	Gene name	Toxic/Non-toxic	Colony color on 100 ng/ml aTc plate*
- Non-toxic control	--	pExTra03	Fruitloop *52* mutant	Non-toxic	+
+ Toxic control	--	pExTra02	Fruitloop *52*	Toxic	-
1	--	pExTra_2	Amelie 2 replicate 1	Non-toxic	+
2	--	pExTra_2	Amelie 2 replicate 2	Non-toxic	+
3	--	pExTra_2	Amelie 2 replicate 3	Non-toxic	+

*Key: NG (no growth) - (no pink color) +(faint pink color) ++(obvious pink color) +++

Gene *3*; Score *0*

Images taken after 4 days at 37 ºC

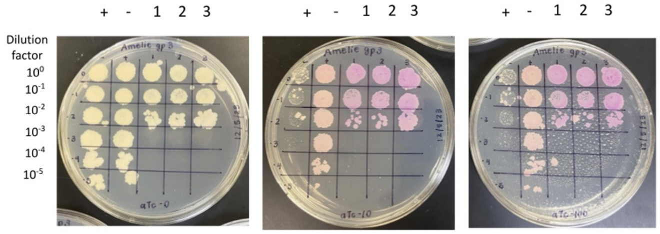

0 ng/ml aTc | 10 ng/ml aTc | 100 ng/ml aTc

Lane	Gene ID	Plasmid name	Gene name	Toxic/Non-toxic	Colony color on 100 ng/ml aTc plate*
- Non-toxic control	--	pExTra03	Fruitloop *52* mutant	Non-toxic	++
+ Toxic control	--	pExTra02	Fruitloop *52*	Toxic	-
1		pExTra-Amelie3	Amelie *3* replicate 1	Non-toxic	+++
2		pExTra-Amelie3	Amelie *3* replicate 2	Non-toxic	+++
3		pExTra-Amelie3	Amelie *3* replicate 3	Non-toxic	+++

*Key: NG (no growth) - (no pink color) +(faint pink color) ++(obvious pink color) +++ (dark pink color)

Gene *4*; Score *0*

Images taken after 4 days at 37 ºC

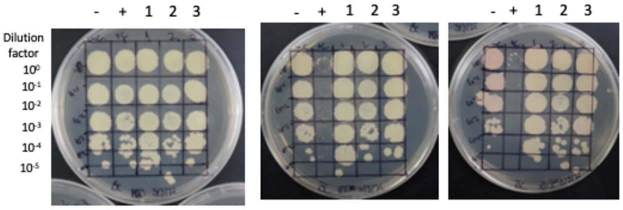

0 ng/ml aTc | 10 ng/ml aTc | 100 ng/ml aTc

Lane	Gene ID	Plasmid name	Gene name	Toxic/Non-toxic	Colony color on 100 ng/ml aTc plate*
- Non-toxic control		pExTra03	Fruitloop *52* mutant	Non-toxic	++
+ Toxic control		pExTra02	Fruitloop *52*	Toxic	-
1		pExTra_4	Amelie 4 replicate 1	Non-toxic	+
2		pExTra_4	Amelie 4 replicate 2	Non-toxic	+
3		pExTra_4	Amelie 4 replicate 3	Non-toxic	+

*Key: NG (no growth) - (no pink color) +(faint pink color) ++(obvious pink color) +++ (dark pink color)

Gene *5*; Score *0*

Images taken after 5 days at 37 ºC

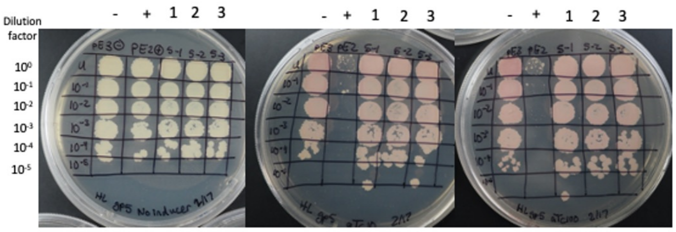

0 ng/ml aTc | 10 ng/ml aTc | 100 ng/ml aTc

Lane	Gene ID	Plasmid name	Gene name	Toxic/Non-toxic	Colony color on 100 ng/ml aTc plate*
- Non-toxic control	--	pExTra03	Fruitloop *52* mutant	Non-toxic	+
+ Toxic control	--	pExTra02	Fruitloop *52*	Toxic	-
1		pExtra_5	Amelie 5 replicate 1	Non-toxic	++
2		pExtra_5	Amelie 5 replicate 2	Non-toxic	++
3		pExtra_5	Amelie 5 replicate 3	Non-toxic	++

*Key: NG (no growth) - (no pink color) +(faint pink color) ++(obvious pink color) +++ (dark pink color)

Gene *7*; Score *0*

Images taken after 5 days at 37˚C

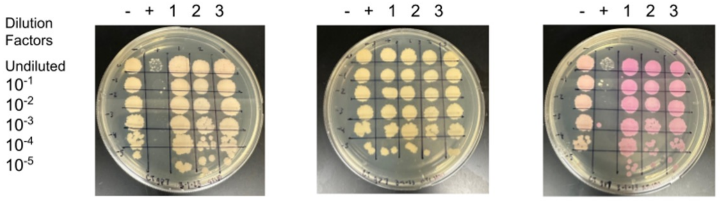

0 ng/ml aTc | 10 ng/ml aTc | 100 ng/ml aTc

Lane	Gene ID	Plasmid Name	Gene Name	Toxic/Non-Toxic	Colony Color on 100 ng/mL aTc Plate
(-) Non-Toxic Control	-----	pExTra_03	Fruitloop *52* Mutant	Non-Toxic	+
(+) Toxic Control	-----	pExTra_02	Fruitloop *52*	Toxic	-
1	-----	pExTra-Amelie_7	Amelie 7 Replicate 1	Non-Toxic	+++
2	-----	pExTra-Amelie_7	Amelie 7 Replicate 2	Non-Toxic	+++
3	-----	pExTra-Amelie_7	Amelie 7 Replicate 3	Non-Toxic	+++

*Key: NG (No Growth) - (no pink color) + (faint pink color) ++ (obvious pink color) +++ (dark pink color)

Gene *8*; Score *1*

Images taken after 5 days at 37 ºC

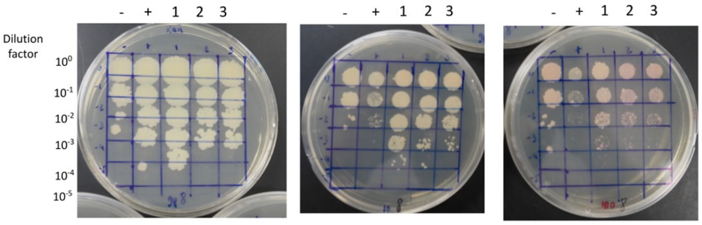

0 ng/ml aTc | 10 ng/ml aTc | 100 ng/ml aTc

Lane	Gene ID	Plasmid name	Gene name	Toxic/Non-toxic	Colony color on 100 ng/ml aTc plate*
- Non-toxic control	--	pExTra03	Fruitloop *52* mutant	Non-toxic	+
+ Toxic control	--	pExTra02	Fruitloop *52*	Toxic	-
1	--	pExTra-Amelie 8	Amelie *8* replicate 1	Toxic	++
2	--	pExTra-Amelie 8	Amelie *8* replicate 2	Toxic	++
3	--	pExTra-Amelie 8	Amelie *8* replicate 3	Toxic	++

*Key: NG (no growth) - (no pink color) +(faint pink color) ++(obvious pink color) +++ (dark pink color)

Gene *9*; Score *3*

Images taken after 4 days at 37 ºC

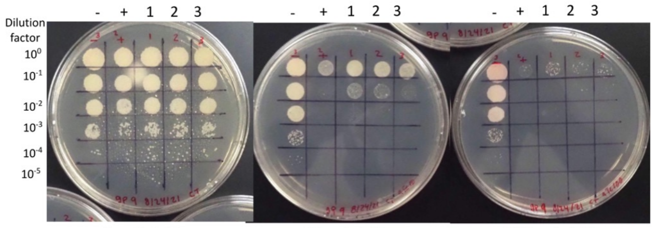

0 ng/ml aTc | 10 ng/ml aTc | 100 ng/ml aTc

Lane	Gene ID	Plasmid name	Gene name	Toxic/Non-toxic	Colony color on 100 ng/ml aTc plate*
- Non-toxic control	--	pExTra03	Fruitloop *52* mutant	Non-toxic	+
+ Toxic control	--	pExTra02	Fruitloop *52*	Toxic	-
1		pExTra-Amelie*09*	Amelie *09* replicate 1	Toxic	++
2		pExTra-Amelie09	Amelie *09* replicate 2	Toxic	++
3		pExTra-Amelie09	Amelie *09* replicate 3	Toxic	++

*Key: NG (no growth) - (no pink color) +(faint pink color) ++(obvious pink color) +++ (dark pink color)

Gene *10*; Score *0*

Images taken after 5 days at 37 ºC

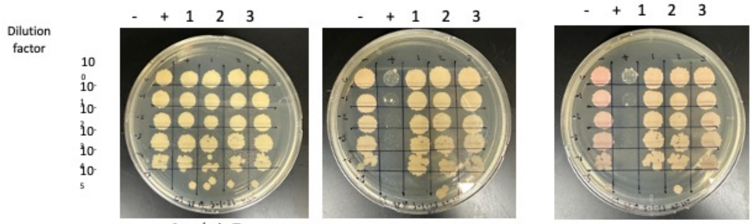

0 ng/ml aTc   10 ng/ml aTc   100 ng/ml aTc

Lane	Gene ID	Plasmid name	Gene name	Toxic/Non-toxic	Colony color on 100 ng/ml aTc plate*
- Non-toxic control	--	pExTra03	Fruitloop *52* mutant	Non-toxic	++
+ Toxic control	--	pExTra02	Fruitloop *52*	Toxic	-
1	--	pExTra_10	Amelie *10* replicate 1	Non-toxic	++
2	--	pExTra_10	Amelie *10* replicate 2	Non-toxic	++
3	--	pExTra_10	Amelie *10* replicate 3	Non-toxic	++

*Key: NG (no growth) - (no pink color) +(faint pink color) ++(obvious pink color) +++ (dark pink color)

Gene *14*; Score *0*

Images taken after 4 days at 37 ºC

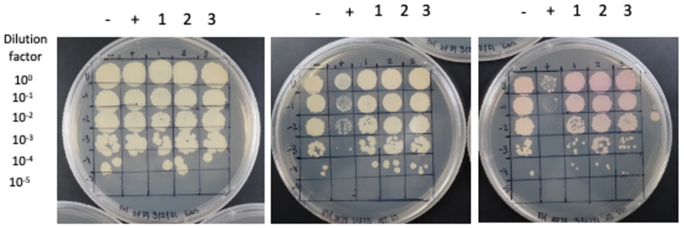

0 ng/ml aTc   10 ng/ml aTc   100 ng/ml aTc

Lane	Gene ID	Plasmid name	Gene name	Toxic/Non-toxic	Colony color on 100 ng/ml aTc plate*
- Non-toxic control	--	pExTra03	Fruitloop *52* mutant	Non-toxic	+
+ Toxic control	--	pExTra02	Fruitloop *52*	Toxic	-
1	--	pExTra-Amelie 14	Amelie 14 replicate 1	Non-toxic	++
2	--	pExTra-Amelie 14	Amelie 14 replicate 2	Non-toxic	++
3	--	pExTra-Amelie 14	Amelie 14 replicate 3	Non-toxic	++

*Key: NG (no growth) - (no pink color) +(faint pink color)++(obvious pink color) +++ (dark pink color)

Gene *11*; Score *0*

Images taken after 5 days at 37 ºC

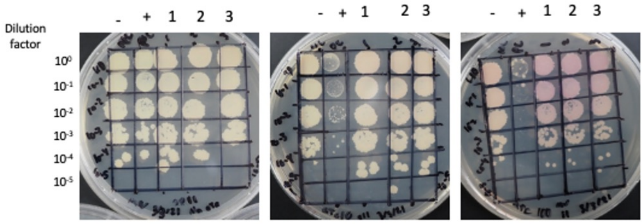

0 ng/ml aTc   10 ng/ml aTc   100 ng/ml aTc

Lane	Gene ID	Plasmid name	Gene name	Toxic/Non-toxic	Colony color on 100 ng/ml aTc plate*
- Non-toxic control	--	pExTra03	Fruitloop *52* mutant	Non-toxic	+
+ Toxic control	--	pExTra02	Fruitloop *52*	Toxic	-
1	--	pExTra_11	Amelie *11* replicate 1	Non-toxic	++
2	--	pExTra_11	Amelie *11* replicate 2	Non-toxic	++
3	--	pExTra_11	Amelie *11* replicate 3	Non-toxic	++

*Key: NG (no growth) - (no pink color) +(faint pink color) ++(obvious pink color) +++ (dark pink color)

Gene *15*; Score *2*

Images taken after 4 days at 37 ºC

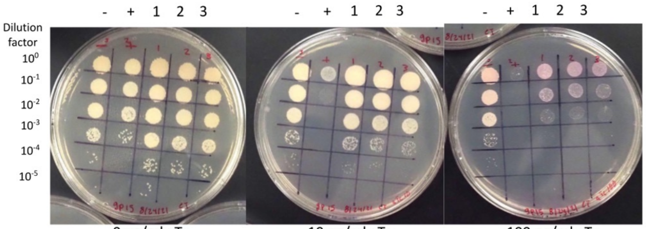

0 ng/ml aTc   10 ng/ml aTc   100 ng/ml aTc

Lane	Gene ID	Plasmid name	Gene name	Toxic/Non-toxic	Colony color on 100 ng/ml aTc plate*
- Non-toxic control	--	pExTra03	Fruitloop *52* mutant	Non-toxic	+
+ Toxic control	--	pExTra02	Fruitloop *52*	Toxic	-
1		pExTra-Amelie15	Amelie 15 replicate 1	Toxic	+++
2		pExTra-Amelie15	Amelie 15 replicate 2	Toxic	+++
3		pExTra-Amelie15	Amelie 15 replicate 3	Toxic	+++

*Key: NG (no growth) - (no pink color) +(faint pink color) ++(obvious pink color) +++ (dark pink color)

Gene *12*; Score *0*

Images taken after 5 days at 37 ºC

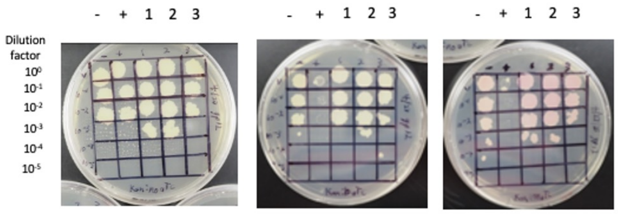

0 ng/ml aTc   10 ng/ml aTc   100 ng/ml aTc

Lane	Gene ID	Plasmid name	Gene name	Toxic/Non-toxic	Colony color on 100 ng/ml aTc plate*
- Non-toxic control	--	pExTra03	Fruitloop *52* mutant	Non-toxic	+
+ Toxic control	--	pExTra02	Fruitloop *52*	Toxic	-
1	--	pExTra_12	Amelie *12* replicate 1	Non-toxic	+
2	--	pExTra_12	Amelie *12* replicate 2	Non-toxic	+
3	--	pExTra_12	Amelie *12* replicate 3	Non-toxic	+

*Key: NG (no growth) - (no pink color) +(faint pink color) ++(obvious pink color) +++ (dark pink color)

Gene *16*; Score *1*

Images taken after 4 days at 37 ºC

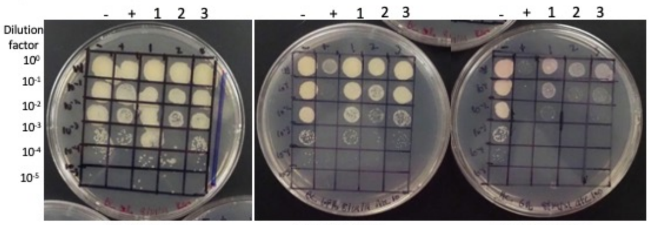

0 ng/ml aTc   10 ng/ml aTc   100 ng/ml aTc

Lane	Gene ID	Plasmid name	Gene name	Toxic/Non-toxic	Colony color on 100 ng/ml aTc plate*
- Non-toxic control	--	pExTra03	Fruitloop *52* mutant	Non-toxic	+
+ Toxic control	--	pExTra02	Fruitloop *52*	Toxic	-
1		pExTra-Amelie16	Amelie 16 replicate 1	Toxic	++
2		pExTra-Amelie16	Amelie 16 replicate 2	Toxic	++
3		pExTra-Amelie16	Amelie 16 replicate 3	Toxic	++

*Key: NG (no growth) - (no pink color) +(faint pink color) ++(obvious pink color) +++ (dark pink color)

Gene *13*; Score *0*

Images taken after 5 days at 37 ºC

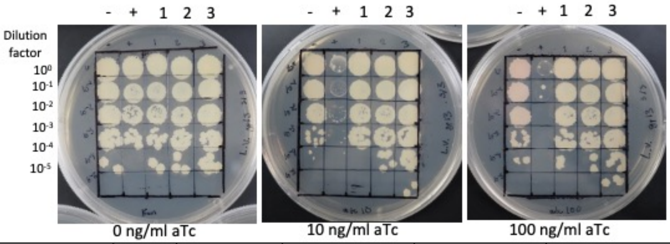

0 ng/ml aTc   10 ng/ml aTc   100 ng/ml aTc

Lane	Gene ID	Plasmid name	Gene name	Toxic/Non-toxic	Colony color on 100 ng/ml aTc plate*
- Non-toxic control	--	pExTra03	Fruitloop *52* mutant	Non-toxic	-
+ Toxic control	--	pExTra02	Fruitloop *52*	Toxic	NG
1	--	pExTra__13	Amelie 13 replicate 1	Non-toxic	-
2	--	pExTra__13	Amelie 13 replicate 1	Non-toxic	-
3	--	pExTra__13	Amelie 13 replicate 1	Non-toxic	-

*Key: NG (no growth) - (no pink color) +(faint pink color) ++(obvious pink color) +++ (dark pink color)

Gene *17*; Score *3*

Images taken after 5 days at 37 ºC

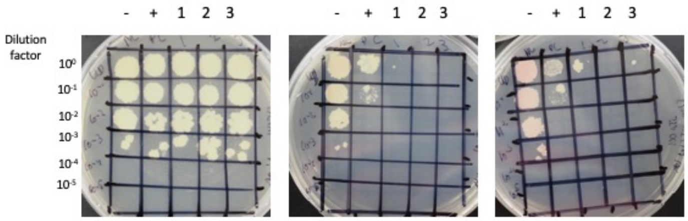

0 ng/ml aTc   10 ng/ml aTc   100 ng/ml aTc

Lane	Gene ID	Plasmid name	Gene name	Toxic/Non-toxic	Colony color on 100 ng/ml aTc plate*
- Non-toxic control	--	pExTra03	Fruitloop *52* mutant	Non-toxic	+
+ Toxic control	--	pExTra02	Fruitloop *52*	Toxic	-
1	--	pExTra-Amelie 17	Amelie *17* replicate 1	Toxic	-
2	--	pExTra-Amelie 17	Amelie *17* replicate 2	Toxic	NG
3	--	pExTra-Amelie 17	Amelie *17* replicate 3	Toxic	NG

*Key: NG (no growth) - (no pink color) +(faint pink color) ++(obvious pink color) +++ (dark pink color)

Gene *18*; Score *0*

Images taken after 5 days at 37 ºC

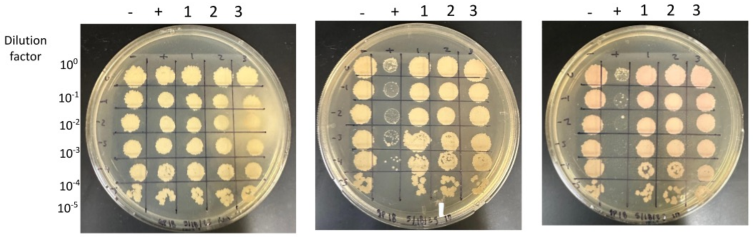

0 ng/ml aTc | 10 ng/ml aTc | 100 ng/ml aTc

Lane	Gene ID	Plasmid name	Gene name	Toxic/Non-toxic	Colony color on 100 ng/ml aTc plate*
- Non-toxic control	--	pExTra03	Fruitloop *52* mutant	Non-toxic	+
+ Toxic control	--	pExTra02	Fruitloop *52*	Toxic	-
1	--	pExTra-Amelie 18	Amelie 18 replicate 1	Non-toxic	++
2	--	pExTra-Amelie 18	Amelie 18 replicate 2	Non-toxic	++
3	--	pExTra-Amelie 18	Amelie 18 replicate 3	Non-toxic	++

*Key: NG (no growth) - (no pink color) +(faint pink color) ++(obvious pink color) +++ (dark pink color)

Gene *19*; Score *0*

Images taken after 5 days at 37 ºC

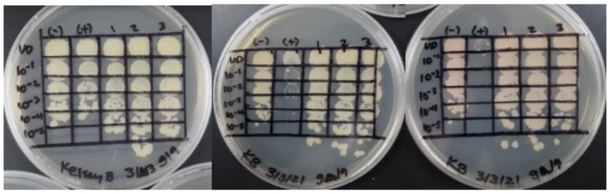

0 ng/ml aTc | 10 ng/ml aTc | 100 ng/ml aTc

Lane	Gene ID	Plasmid name	Gene name	Toxic/Non-toxic	Colony color on 100 ng/ml aTc plate*
- Non-toxic control	--	pExTra03	Fruitloop *52* mutant	Non-toxic	+
+ Toxic control	--	pExTra02	Fruitloop *52*	Toxic	-
1	--	pExTra_19	Amelie 19 replicate 1	Non-toxic	+
2	--	pExTra_19	Amelie 19 replicate 2	Non-toxic	+
3	--	pExTra_19	Amelie 19 replicate 3	Non-toxic	+

*Key: NG (no growth) - (no pink color) +(faint pink color) ++(obvious pink color) +++ (dark pink color)

Gene *20*; Score 2

Images taken after 4 days at 37 ºC

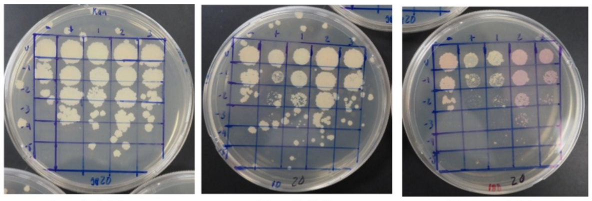

0 ng/ml aTc | 10 ng/ml aTc | 100 ng/ml aTc

Lane	Gene ID	Plasmid name	Gene name	Toxic/Non-toxic	Colony color on 100 ng/ml aTc plate*
- Non-toxic control	--	pExTra03	Fruitloop *52* mutant	Non-toxic	+
+ Toxic control	--	pExTra02	Fruitloop *52*	Toxic	-
1		pExTra-Amelie20	Amelie 20 replicate 1	Toxic	++
2		pExTra-Amelie20	Amelie 20 replicate 2	Toxic	++
3		pExTra-Amelie20	Amelie 20 replicate 3	Toxic	++

*Key: NG (no growth) - (no pink color) +(faint pink color) ++(obvious pink color) +++ (dark pink color)

Gene *21*; Score *0*

Images taken after 5 days at 37 ºC

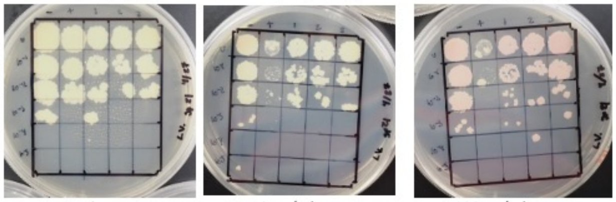

0 ng/ml aTc | 10 ng/ml aTc | 100 ng/ml aTc

Lane	Gene ID	Plasmid name	Gene name	Toxic/Non-toxic	Colony color on 100 ng/ml aTc plate*
- Non-toxic control	--	pExTra03	Fruitloop *52* mutant	Non-toxic	+
+ Toxic control	--	pExTra02	Fruitloop *52*	Toxic	-
1	--	pExTra__Amelie21	Amelie 21 replicate 1	Non-toxic	++
2	--	pExTra__Amelie21	Amelie 21 replicate 2	Non-toxic	++
3	--	pExTra__Amelie21	Amelie 21 replicate 3	Non-toxic	++

*Key: NG (no growth) - (no pink color) +(faint pink color) ++(obvious pink color) +++ (dark pink color)

Gene *22*; Score *0*

Images taken after 5 days at 37 ºC

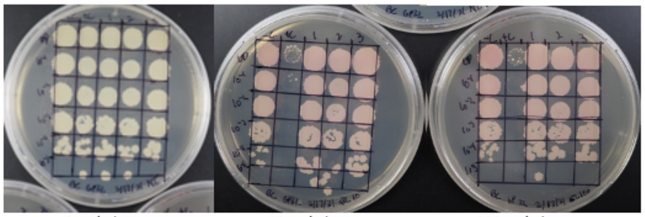

0 ng/ml aTc | 10 ng/ml aTc | 100 ng/ml aTc

Lane	Gene ID	Plasmid name	Gene name	Toxic/Non-toxic	Colony color on 100 ng/ml aTc plate*
- Non-toxic control		pExTra03	Fruitloop *52* mutant	Non-toxic	++
+ Toxic control		pExTra02	Fruitloop *52*	Toxic	-
1		pExTra_22	Amelie *22* replicate 1	Non-toxic	++
2		pExTra_22	Amelie *22* replicate 2	Non-toxic	++
3		pExTra_22	Amelie *22* replicate 3	Non-toxic	++

*Key: NG (no growth) - (no pink color) +(faint pink color) ++(obvious pink color) +++ (dark pink color)

Gene *23*; Score *0*

Images taken after 5 days at 37 ºC

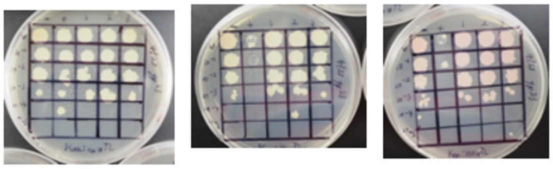

0 ng/ml aTc | 10 ng/ml aTc | 100 ng/ml aTc

Lane	Gene ID	Plasmid name	Gene name	Toxic/Non-toxic	Colony color on 100 ng/ml aTc plate*
- Non-toxic control	--	pExTra03	Fruitloop *52* mutant	Non-toxic	+
+ Toxic control	--	pExTra02	Fruitloop *52*	Toxic	-
1	--	pExTra_23	Amelie *23* replicate 1	Non-toxic	+
2	--	pExTra_23	Amelie *23* replicate 2	Non-toxic	+
3	--	pExTra_23	Amelie *23* replicate 3	Non-toxic	+

*Key: NG (no growth) - (no pink color) +(faint pink color) ++(obvious pink color) +++ (dark pink color)

Gene *24*; Score *0*

Images taken after 5 days at 37 ºC

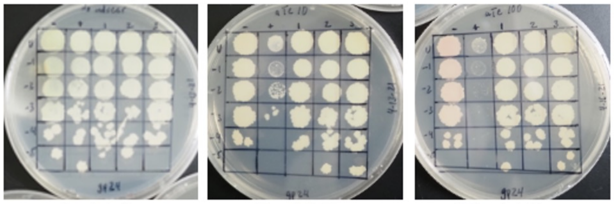

0 ng/ml aTc | 10 ng/ml aTc | 100 ng/ml aTc

Lane	Gene ID	Plasmid name	Gene name	Toxic/Non-toxic	Colony color on 100 ng/ml aTc plate*
- Non-toxic control	--	pExTra03	Fruitloop *52* mutant	Non-toxic	+
+ Toxic control	--	pExTra02	Fruitloop *52*	Toxic	-
1		pExTra- Amelie 24	Amelie 24 replicate 1	Non-toxic	-
2		pExTra- Amelie 24	Amelie 24 replicate 2	Non-toxic	-
3		pExTra- Amelie 24	Amelie 24 replicate 3	Non-toxic	-

*Key: NG (no growth) - (no pink color) +(faint pink color) ++(obvious pink color) +++ (dark pink color)

Gene *25*; Score *0*

Images taken after 5 days at 37°C

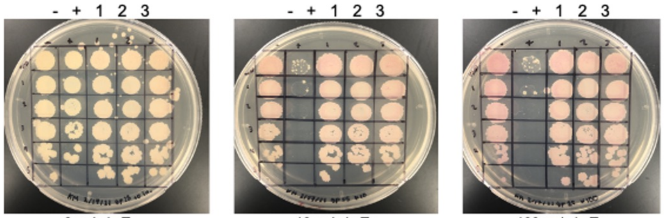

0 ng/ml aTc | 10 ng/ml aTc | 100 ng/ml aTc

Lane	Gene ID	Plasmid Name	Gene Name	Toxic/Non-Toxic	Colony Color on 100 ng/mL aTc Plate
(-) Non-Toxic Control	-----	pExTra_03	Fruitloop *52* Mutant	Non-Toxic	++
(+) Toxic Control	-----	pExTra_02	Fruitloop *52*	Toxic	-
1	-----	pExTra_25	Amelie 25 Replicate 1	Non-Toxic	++
2	-----	pExTra_25	Amelie 25 Replicate 2	Non-Toxic	++
3	-----	pExTra_25	Amelie 25 Replicate 3	Non-Toxic	++

*Key: NG (No Growth) - (no pink color) + (faint pink color) ++ (obvious pink color) +++ (dark pink color)

Gene *26*; Score *0*

Images taken after 5 days at 37 ºC

Dilution factor: 10^{0}, 10^{-1}, 10^{-2}, 10^{-3}, 10^{-4}, 10^{-5}

Lanes: - + 1 2 3 | - + 1 2 3 | - + 1 2 3

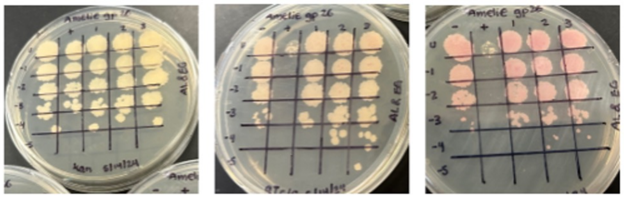

0 ng/ml aTc** | 10 ng/ml aTc | 100 ng/ml aTc

Lane	Gene ID	Plasmid name	Gene name	Toxic/Non-toxic	Colony color on 100 ng/ml aTc plate*
- Non-toxic control	--	pExTra03	Fruitloop *52* mutant	Non-toxic	+
+ Toxic control	--	pExTra02	Fruitloop *52*	Toxic	+
1	--	pExTra_Amelie26	Amelie *26* replicate 1	Non-toxic	+
2	--	pExTra_Amelie26	Amelie *26* replicate 2	Non-toxic	+
3	--	pExTra_Amelie26	Amelie *26* replicate 3	Non-toxic	+

*Key: NG (no growth) - (no pink color) +(faint pink color) ++(obvious pink color) +++ (dark pink color)

Gene *27*; Score *0*

Images taken after 5 days at 37 ºC

Dilution factor: 10^{0}, 10^{-1}, 10^{-2}, 10^{-3}, 10^{-4}, 10^{-5}

Lanes: - + 1 2 3 | - + 1 2 3 | - + 1 2 3

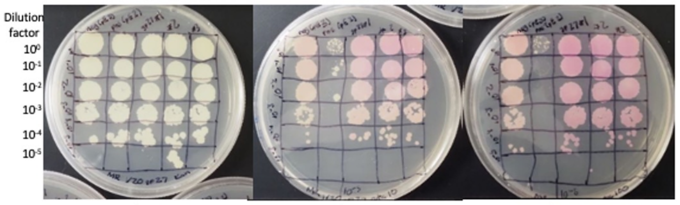

0 ng/ml aTc | 10 ng/ml aTc | 100 ng/ml aTc

Lane	Gene ID	Plasmid name	Gene name	Toxic/Non-toxic	Colony color on 100 ng/ml aTc plate*
- Non-toxic control	--	pExTra03	Fruitloop *52* mutant	Non-toxic	+
+ Toxic control	--	pExTra02	Fruitloop *52*	Toxic	-
1		pExtra_27	Amelie 27 replicate 1	Non-toxic	++
2		pExtra_27	Amelie 27 replicate 2	Non-toxic	++
3		pExtra_27	Amelie 27 replicate 3	Non-toxic	++

*Key: NG (no growth) - (no pink color) +(faint pink color) ++(obvious pink color) +++ (dark pink color)

Gene *28*; Score *0*

Images taken after 5 days at 37 ºC

Dilution factor: 10^{0}, 10^{-1}, 10^{-2}, 10^{-3}, 10^{-4}, 10^{-5}

Lanes: - + 1 2 3 | - + 1 2 3 | - + 1 2 3

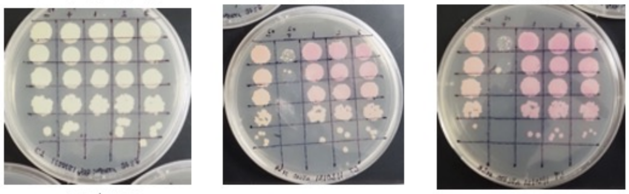

0 ng/ml aTc | 10 ng/ml aTc | 100 ng/ml aTc

Lane	Gene ID	Plasmid name	Gene name	Toxic/Non-toxic	Colony color on 100 ng/ml aTc plate*
- Non-toxic control	--	pExTra03	Fruitloop *52* mutant	Non-toxic	++
+ Toxic control	--	pExTra02	Fruitloop *52*	Toxic	-
1	--	pExTra_Amelie28	Amelie *28* replicate 1	Non-toxic	++
2	--	pExTra_Amelie28	Amelie *28* replicate 2	Non-toxic	++
3	--	pExTra_Amelie28	Amelie *28* replicate 3	Non-toxic	++

*Key: NG (no growth.)- (no pink color) +(faint pink color) ++(obvious pink color). +++ (dark pink color)

Gene *29*; Score *3*

Images taken after 5 days at 37 ºC

Dilution factor: 10^{0}, 10^{-1}, 10^{-2}, 10^{-3}, 10^{-4}, 10^{-5}

Lanes: - + 1 2 3 | - + 1 2 3 | - + 1 2 3

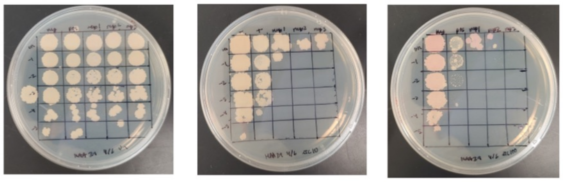

0 ng/ml aTc** | 10 ng/ml aTc | 100 ng/ml aTc

Lane	Gene ID	Plasmid name	Gene name	Toxic/Non-toxic	Colony color on 100 ng/ml aTc plate*
- Non-toxic control	--	pExTra03	Fruitloop *52* mutant	Non-toxic	+
+ Toxic control	--	pExTra02	Fruitloop *52*	Toxic	+
1	--	pExTra_29	Amelie *29* replicate 1	Toxic	+
2	--	pExTra_29	Amelie *29* replicate 2	Toxic	+
3	--	pExTra_29	Amelie *29* replicate 3	Toxic	NG

*Key: NG (no growth) - (no pink color) +(faint pink color) ++(obvious pink color) +++ (dark pink color)

Gene *30*; Score *0*

Images taken after 4 days at 37 ºC

Dilution factor: 10^{0}, 10^{-1}, 10^{-2}, 10^{-3}, 10^{-4}, 10^{-5}

Lanes: - + 1 2 3 | - + 1 2 3 | - + 1 2 3

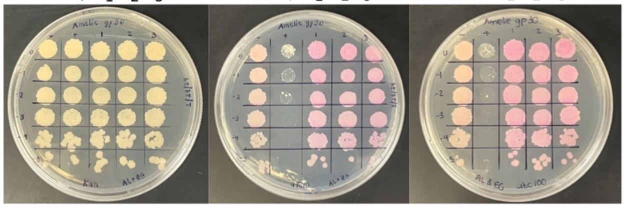

0 ng/ml aTc | 10 ng/ml aTc | 100 ng/ml aTc

Lane	Gene ID	Plasmid name	Gene name	Toxic/Non-toxic	Colony color on 100 ng/ml aTc plate*
+ Toxic control	--	pExTra02	Fruitloop *52*	Toxic	-
- Non-toxic control	--	pExTra03	Fruitloop *52* mutant	Non-toxic	+
1	131436	pExTra-Amelie30	Amelie *30* replicate 1	Non-toxic	++
2	131436	pExTra-Amelie30	Amelie *30* replicate 2	Non-toxic	++
3	131436	pExTra-Amelie30	Amelie *30* replicate 3	Non-toxic	++

*Key: NG (no growth) - (no pink color) +(faint pink color) ++(obvious pink color) +++ (dark pink color)

Gene *31*; Score *2*

Images taken after 5 days at 37 ºC

Dilution factor: 10^{0}, 10^{-1}, 10^{-2}, 10^{-3}, 10^{-4}, 10^{-5}

Lanes: - + 1 2 3 | - + 1 2 3 | - + 1 2 3

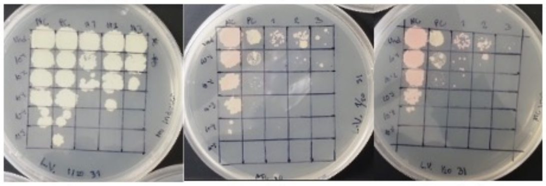

0 ng/ml aTc | 10 ng/ml aTc | 100 ng/ml aTc

Lane	Gene ID	Plasmid name	Gene name	Toxic/Non-toxic	Colony color on 100 ng/ml aTc plate*
- Non-toxic control	--	pExTra03	Fruitloop *52* mutant	Non-toxic	++
+ Toxic control	--	pExTra02	Fruitloop *52*	Toxic	-
1	--	pExTra__Amelie31	Amelie 31 replicate 1	Toxic	+
2	--	pExTra__Amelie31	Amelie 31 replicate 2	Toxic	+
3	--	pExTra__Amelie31	Amelie 31 replicate 3	Toxic	NG

*Key: NG (no growth) - (no pink color) +(faint pink color) ++(obvious pink color) +++ (dark pink color)

Gene *32*; Score *2*

Images taken after 4 days at 37 ºC

Dilution factor: 10^{0}, 10^{-1}, 10^{-2}, 10^{-3}, 10^{-4}, 10^{-5}

Lanes: - + 1 2 3 | - + 1 2 3 | - + 1 2 3

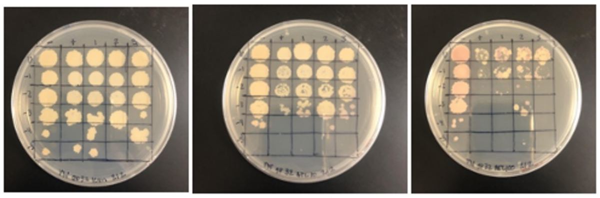

0 ng/ml aTc | 10 ng/ml aTc | 100 ng/ml aTc

Lane	Gene ID	Plasmid name	Gene name	Toxic/Non-toxic	Colony color on 100 ng/ml aTc plate*
- Non-toxic control	--	pExTra03	Fruitloop *52* mutant	Non-toxic	++
+ Toxic control	--	pExTra02	Fruitloop *52*	Toxic	-
1	--	pExTra-Amelie 32	Amelie 32 replicate 1	toxic	+
2	--	pExTra-Amelie 32	Amelie 32 replicate 2	toxic	+
3	--	pExTra-Amelie 32	Amelie 32 replicate 3	toxic	+

*Key: NG (no growth) - (no pink color) +(faint pink color)++(obvious pink color) +++ (dark pink color)

Gene *33*; Score *0*

Images taken after 5 days at 37 ºC

Dilution factor: 10^{0}, 10^{-1}, 10^{-2}, 10^{-3}, 10^{-4}, 10^{-5}

Lanes: + - 1 2 3 | + - 1 2 3 | + - 1 2 3

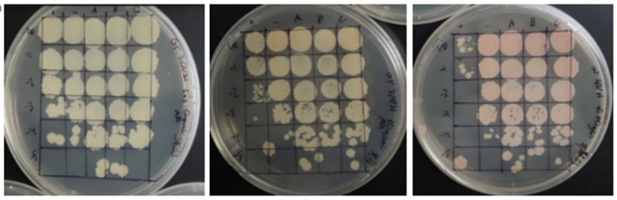

0 ng/ml aTc | 10 ng/ml aTc | 100 ng/ml aTc

Lane	Gene ID	Plasmid name	Gene name	Toxic/Non-toxic	Colony color on 100 ng/ml aTc plate*
- Non-toxic control	--	pExTra03	Fruitloop *52* mutant	Non-toxic	++
+ Toxic control	--	pExTra02	Fruitloop *52*	Toxic	+
1	--	pExTra_33	Amelie 33 replicate 1	Non-toxic	++
2	--	pExTra_33	Amelie 33 replicate 2	Non-toxic	++
3	--	pExTra_33	Amelie 33 replicate 3	Non-toxic	++

*Key: NG (no growth) - (no pink color) +(faint pink color) ++(obvious pink color) +++ (dark pink color)

Gene *34*; Score *3*

Images taken after 5 days at 37 ºC

- + 1 2 3

Dilution factor 10, 10^{-1}, 10^{-2}, 10^{-3}, 10^{-4}, 10^{-5}

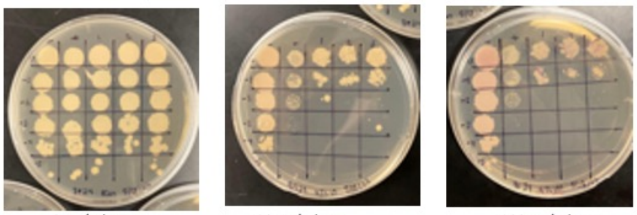

0 ng/ml aTc | 10 ng/ml aTc | 100 ng/ml aTc

Lane	Gene ID	Plasmid name	Gene name	Toxic/Non-toxic	Colony color on 100 ng/ml aTc plate*
- Non-toxic control	--	pExTra03	Fruitloop *52* mutant	Non-toxic	+
+ Toxic control	--	pExTra02	Fruitloop *52*	Toxic	-
1		pExTra-Amelie 34	Amelie 34 replicate 1	Toxic	+++
2		pExTra-Amelie 34	Amelie 34 replicate 2	Toxic	++
3		pExTra-Amelie 34	Amelie 34 replicate 3	Toxic	++

*Key: NG (no growth) - (no pink color) +(faint pink color) ++(obvious pink color) +++ (dark pink color)

Gene *35*; Score *0*

Images taken after 5 days at 37 ºC

Dilution factor 10^{0}, 10^{-1}, 10^{-2}, 10^{-3}, 10^{-4}, 10^{-5}

- + 1 2 3

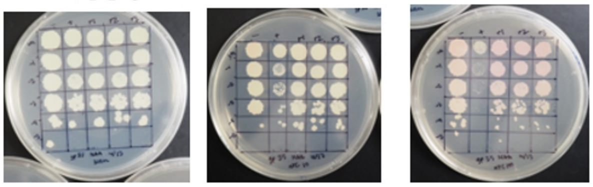

0 ng/ml aTc* | 10 ng/ml aTc | 100 ng/ml aTc

Lane	Gene ID	Plasmid name	Gene name	Toxic/Non-toxic	Colony color on 100 ng/ml aTc plate*
- Non-toxic control	--	pExTra03	Fruitloop *52* mutant	Non-toxic	+
+ Toxic control	--	pExTra02	Fruitloop *52*	Toxic	+
1	--	pExTra_35	Amelie *35* replicate 1	Non-toxic	+
2	--	pExTra_35	Amelie *35* replicate 2	Non-toxic	+
3	--	pExTra_35	Amelie *35* replicate 3	Non-toxic	+

*Key: NG (no growth) - (no pink color) +(faint pink color) ++(obvious pink color) +++ (dark pink color)

Gene *36*; Score *0*

Images taken after 5 days at 37 ºC

- + 1 2 3

Dilution factor 10^{0}, 10^{-1}, 10^{-2}, 10^{-3}, 10^{-4}, 10^{-5}

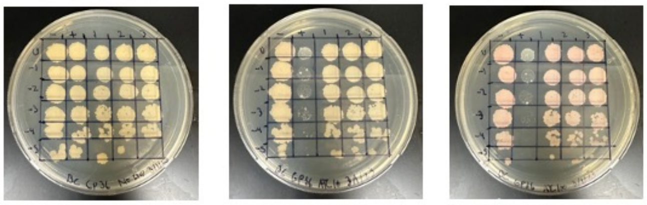

0 ng/ml aTc | 10 ng/ml aTc | 100 ng/ml aTc

Lane	Gene ID	Plasmid name	Gene name	Toxic/Non-toxic	Colony color on 100 ng/ml aTc plate*
- Non-toxic control	--	pExTra03	Fruitloop *52* mutant	Non-toxic	+
+ Toxic control	--	pExTra02	Fruitloop *52*	Toxic	-
1	--	pExTra_36	Amelie 36 replicate 1	Non-toxic	+
2	--	pExTra_36	Amelie 36 replicate 2	Non-toxic	+
3	--	pExTra_36	Amelie 36 replicate 3	Non-toxic	++

*Key: NG (no growth) - (no pink color) +(faint pink color) ++(obvious pink color) +++ (dark pink color)

Gene *37*; Score *0*

Images taken after 4 days at 37 ºC

- + 1 2 3

Dilution factor 10^{0}, 10^{-1}, 10^{-2}, 10^{-3}, 10^{-4}, 10^{-5}

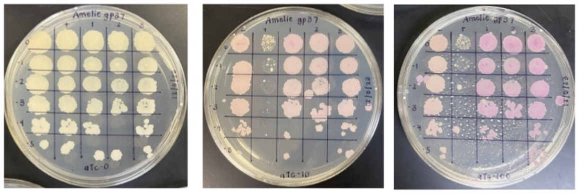

0 ng/ml aTc | 10 ng/ml aTc | 100 ng/ml aTc

Lane	Gene ID	Plasmid name	Gene name	Toxic/Non-toxic	Colony color on 100 ng/ml aTc plate*
- Non-toxic control	--	pExTra03	Fruitloop *52* mutant	Non-toxic	++
+ Toxic control	--	pExTra02	Fruitloop *52*	Toxic	-
1		pExTra-Amelie37	Amelie *37*replicate 1	Non-toxic	+++
2		pExTra-Amelie37	Amelie *37* replicate 2	Non-toxic	+++
3		pExTra-Amelie37	Amelie *37* replicate 3	Non-toxic	+++

*Key: NG (no growth) - (no pink color) +(faint pink color) ++(obvious pink color) +++ (dark pink color)

Gene *38*; Score *0*

Images taken after 4 days at 37 ºC

- + 1 2 3

Dilution factor 10^{0}, 10^{-1}, 10^{-2}, 10^{-3}, 10^{-4}, 10^{-5}

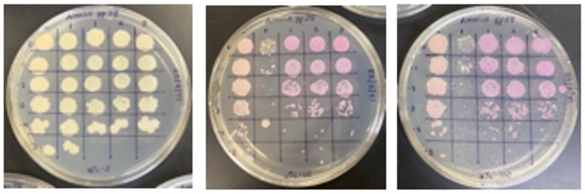

0 ng/ml aTc | 10 ng/ml aTc | 100 ng/ml aTc

Lane	Gene ID	Plasmid name	Gene name	Toxic/Non-toxic	Colony color on 100 ng/ml aTc plate*
- Non-toxic control	--	pExTra03	Fruitloop *52* mutant	Non-toxic	++
+ Toxic control	--	pExTra02	Fruitloop *52*	Toxic	-
1		pExTra-Amelie38	Amelie *38* replicate 1	Non-toxic	+++
2		pExTra-Amelie38	Amelie *38* replicate 2	Non-toxic	+++
3		pExTra-Amelie38	Amelie *38* replicate 3	Non-toxic	+++

*Key: NG (no growth) - (no pink color) +(faint pink color) ++(obvious pink color) +++ (dark pink color)

Gene *39*; Score *0*

Images taken after 5 days at 37 ºC

- + 1 2 3

Dilution factor 10^{0}, 10^{-1}, 10^{-2}, 10^{-3}, 10^{-4}, 10^{-5}

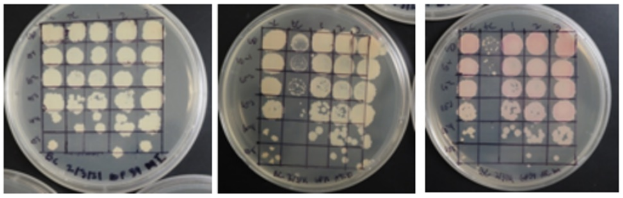

0 ng/ml aTc | 10 ng/ml aTc | 100 ng/ml aTc

Lane	Gene ID	Plasmid name	Gene name	Toxic/Non-toxic	Colony color on 100 ng/ml aTc plate*
- Non-toxic control	--	pExTra03	Fruitloop *52* mutant	Non-toxic	+
+ Toxic control	--	pExTra02	Fruitloop *52*	Toxic	-
1	--	pExTra-39	Amelie 39 replicate 1	Non-toxic	++
2	--	pExTra-39	Amelie 39 replicate 2	Non-toxic	++
3	--	pExTra-39	Amelie 39 replicate 3	Non-toxic	++

*Key: NG (no growth) - (no pink color) +(faint pink color) ++(obvious pink color) +++ (dark pink color)

Gene *40*; Score *0*

Images taken after 5 days at 37 ºC

- + 1 2 3

Dilution factor 10^{0}, 10^{-1}, 10^{-2}, 10^{-3}, 10^{-4}, 10^{-5}

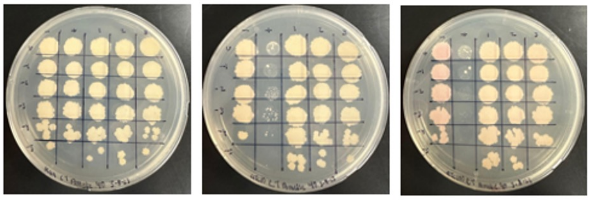

0 ng/ml aTc | 10 ng/ml aTc | 100 ng/ml aTc

Lane	Gene ID	Plasmid name	Gene name	Toxic/Non-toxic	Colony color on 100 ng/ml aTc plate*
- Non-toxic control	--	pExTra03	Fruitloop *52* mutant	Non-toxic	+
+ Toxic control	--	pExTra02	Fruitloop *52*	Toxic	-
1		pExtra_40	Amelie 40 replicate 1	Non-toxic	+
2		pExtra_40	Amelie 40 replicate 2	Non-toxic	+
3		pExtra_40	Amelie 40 replicate 3	Non-toxic	+

*Key: NG (no growth) - (no pink color) +(faint pink color) ++(obvious pink color) +++ (dark pink color)

Gene *41*; Score *0*

Images taken after 5 days at 37ºC

- + 1 2 3

Dilution factor 10^{0}, 10^{-1}, 10^{-2}, 10^{-3}, 10^{-4}, 10^{-5}

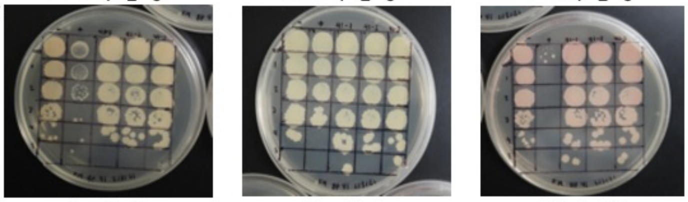

0 ng/ml aTc | 10 ng/ml aTc | 100 ng/ml aTc

Lane	Gene ID	Plasmid Name	Gene Name	Toxic/Non-Toxic	Colony Color on 100 ng/mL aTc Plate
(-) Non-Toxic Control	-----	pExTra_03	Fruitloop *52* Mutant	Non-Toxic	+
(+) Toxic Control	-----	pExTra_02	Fruitloop *52*	Toxic	-
1	-----	pExTra_41	Amelie 41 Replicate 1	Non-Toxic	+
2	-----	pExTra_41	Amelie 41 Replicate 2	Non-Toxic	+
3	-----	pExTra_41	Amelie 41 Replicate 3	Non-Toxic	+

*Key: NG (No Growth) - (no pink color) + (faint pink color) ++ (obvious pink color) +++ (dark pink color)

Gene *42*; Score *0*

Images taken after 5 days at 37 ºC

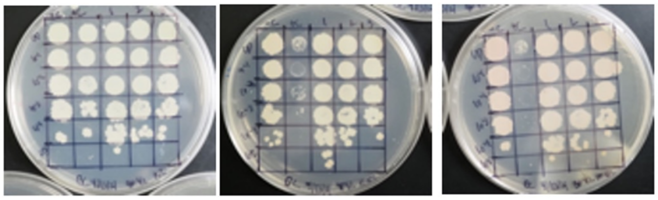

0 ng/ml aTc | 10 ng/ml aTc | 100 ng/ml aTc

Lane	Gene ID	Plasmid name	Gene name	Toxic/Non-toxic	Colony color on 100 ng/ml aTc plate*
- Non-toxic control	--	pExTra03	Fruitloop *52* mutant	Non-toxic	+
+ Toxic control	--	pExTra02	Fruitloop *52*	Toxic	-
1	--	pExTra_42	Amelie *42* replicate 1	Non-toxic	+
2	--	pExTra_42	Amelie *42* replicate 2	Non-toxic	+
3	--	pExTra_42	Amelie *42* replicate 3	Non-toxic	+

*Key: NG (no growth) - (no pink color) +(faint pink color) ++(obvious pink color) +++ (dark pink color)

Gene *43*; Score *3*

Images taken after 5 days at 37 ºC

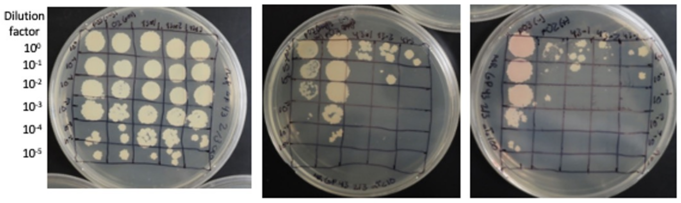

0 ng/ml aTc | 10 ng/ml aTc | 100 ng/ml aTc

Lane	Gene ID	Plasmid name	Gene name	Toxic/Non-toxic	Colony color on 100 ng/ml aTc plate*
- Non-toxic control	--	pExTra03	Fruitloop *52* mutant	Non-toxic	+
+ Toxic control	--	pExTra02	Fruitloop *52*	Toxic	-
1		pExtra_43	Amelie 43 replicate 1	Toxic	-
2		pExtra_43	Amelie 43 replicate 2	Toxic	-
3		pExtra_43	Amelie 43 replicate 3	Toxic	-

*Key: NG (no growth) - (no pink color) +(faint pink color) ++(obvious pink color) +++ (dark pink color)

Gene *44*; Score *3*

Images taken after 4 days at 37 ºC

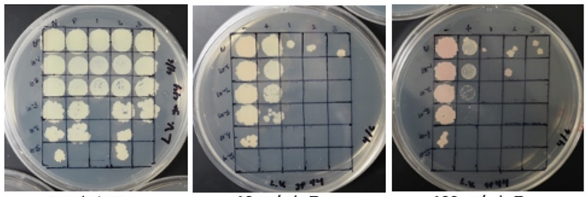

0 ng/ml aTc | 10 ng/ml aTc | 100 ng/ml aTc

Lane	Gene ID	Plasmid name	Gene name	Toxic/Non-toxic	Colony color on 100 ng/ml aTc plate*
- Non-toxic control	--	pExTra03	Fruitloop *52* mutant	Non-toxic	++
+ Toxic control	--	pExTra02	Fruitloop *52*	Toxic	-
1	--	pExTra__44	Amelie 44 replicate 1	Toxic	NG
2	--	pExTra__44	Amelie 44 replicate 1	Toxic	+
3	--	pExTra__44	Amelie 44 replicate 1	Toxic	+

*Key: NG (no growth) - (no pink color) +(faint pink color) ++(obvious pink color) +++

Gene *45*; Score *0*

Images taken after 5 days at 37 ºC

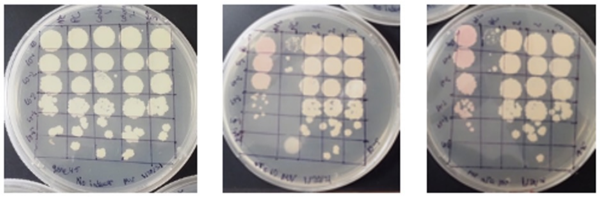

0 ng/ml aTc | 10 ng/ml aTc | 100 ng/ml aTc

Lane	Gene ID	Plasmid name	Gene name	Toxic/Non-toxic	Colony color on 100 ng/ml aTc plate*
- Non-toxic control	--	pExTra03	Fruitloop *52* mutant	Non-toxic	++
+ Toxic control	--	pExTra02	Fruitloop *52*	Toxic	-
1	--	pExTra_45	Amelie *45* replicate 1	Non-toxic	+
2	--	pExTra_45	Amelie *45* replicate 2	Non-toxic	+
3	--	pExTra_45	Amelie *45* replicate 3	Non-toxic	+

*Key: NG (no growth) - (no pink color) +(faint pink color) ++(obvious pink color) +++ (dark pink color)

Gene *46*; Score *1*

Images taken after 5 days at 37 ºC

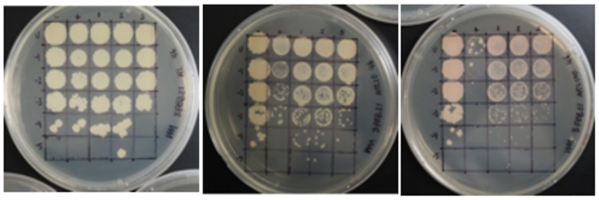

0 ng/ml aTc | 10 ng/ml aTc | 100 ng/ml aTc

Lane	Gene ID	Plasmid name	Gene name	Toxic/Non-toxic	Colony color on 100 ng/ml aTc plate*
- Non-toxic control	--	pExTra03	Fruitloop *52* mutant	Non-toxic	++
+ Toxic control	--	pExTra02	Fruitloop *52*	Toxic	+
1	--	pExTra_46	Amelie *46* replicate 1	toxic	+
2	--	pExTra_46	Amelie *46* replicate 2	toxic	+
3	--	pExTra_46	Amelie *46* replicate 3	toxic	+

*Key: NG (no growth) - (no pink color) +(faint pink color) ++(obvious pink color) +++ (dark pink color)

Gene *47*; Score *3*

Images taken after 5 days at 37 ºC

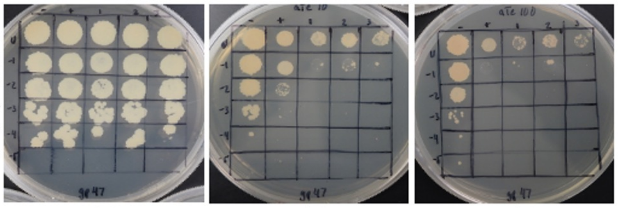

0 ng/ml aTc | 10 ng/ml aTc | 100 ng/ml aTc

Lane	Gene ID	Plasmid name	Gene name	Toxic/Non-toxic	Colony color on 100 ng/ml aTc plate*
- Non-toxic control	--	pExTra03	Fruitloop *52* mutant	Non-toxic	+
+ Toxic control	--	pExTra02	Fruitloop *52*	Toxic	-
1		pExTra- Amelie 47	Amelie 47 replicate 1	Toxic	-
2		pExTra- Amelie 47	Amelie 47 replicate 2	Toxic	-
3		pExTra- Amelie 47	Amelie 47replicate 3	Toxic	-

*Key: NG (no growth) - (no pink color) +(faint pink color) ++(obvious pink color) +++ (dark pink color)

Gene *48*; Score *0*

Images taken after 4 days at 37 ºC

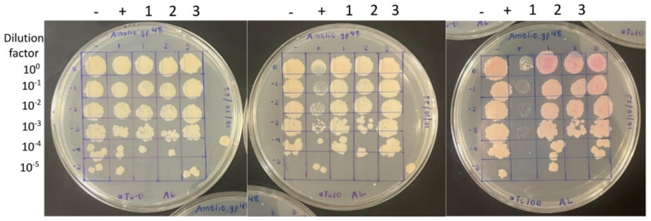

0 ng/ml aTc | 10 ng/ml aTc | 100 ng/ml aTc

Lane	Gene ID	Plasmid name	Gene name	Toxic/Non-toxic	Colony color on 100 ng/ml aTc plate*
+ Toxic control	--	pExTra02	Fruitloop *52*	Toxic	-
- Non-toxic control	--	pExTra03	Fruitloop *52* mutant	Non-toxic	+
1	131436	pExTra-Amelie48	Amelie *48* replicate 1	Non-toxic	++
2	131436	pExTra-Amelie48	Amelie *48* replicate 2	Non-toxic	++
3	131436	pExTra-Amelie48	Amelie *48* replicate 3	Non-toxic	++

*Key: NG (no growth) - (no pink color) +(faint pink color) ++(obvious pink color) +++ (dark pink color)

Gene *49*; Score *3*

Images taken after 4 days at 37 ºC

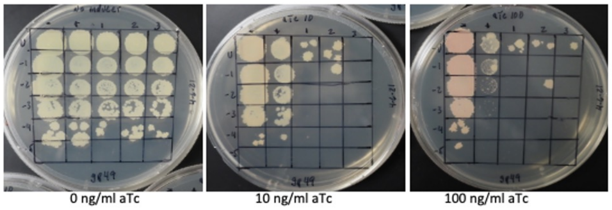

0 ng/ml aTc | 10 ng/ml aTc | 100 ng/ml aTc

Lane	Gene ID	Plasmid name	Gene name	Toxic/Non-toxic	Colony color on 100 ng/ml aTc plate*
- Non-toxic control	--	pExTra03	Fruitloop *52* mutant	Non-toxic	+
+ Toxic control	--	pExTra02	Fruitloop *52*	Toxic	-
1	--	pExTra-Amelie 49	Amelie 49 replicate 1	Toxic	-
2	--	pExTra-Amelie 49	Amelie 49 replicate 2	Toxic	-
3	--	pExTra-Amelie 49	Amelie 49 replicate 3	Toxic	-

*Key: NG (no growth) - (no pink color) +(faint pink color)++(obvious pink color) +++ (dark pink color)

Gene *50*; Score *2*

Images taken after 5 days at 37 ºC

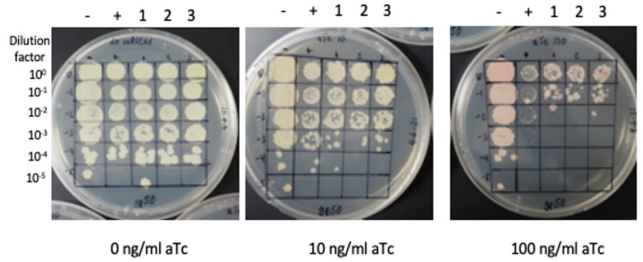

Lane	Gene ID	Plasmid name	Gene name	Toxic/Non-toxic	Colony color on 100 ng/ml aTc plate*
- Non-toxic control	--	pExTra03	Fruitloop *52* mutant	Non-toxic	++
+ Toxic control	--	pExTra02	Fruitloop *52*	Toxic	+
1	--	pExTra_50	Amelie *50* replicate 1	Toxic	+
2	--	pExTra_50	Amelie *50* replicate 2	Toxic	+
3	--	pExTra_50	Amelie *50* replicate 3	Toxic	+

*Key: NG (no growth) - (no pink color) +(faint pink color) ++(obvious pink color) +++ (dark pink color)

Gene *51*; Score *0*

Images taken after 5 days at 37 ºC

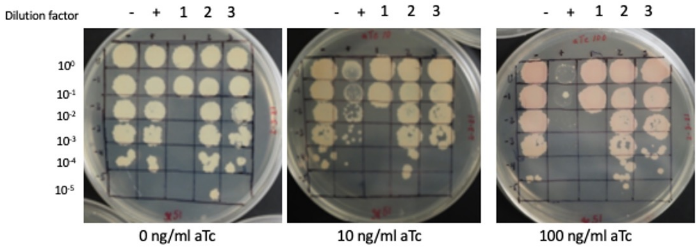

Lane	Gene ID	Plasmid name	Gene name	Toxic/Non-toxic	Colony color on 100 ng/ml aTc plate*
- Non-toxic control	--	pExTra03	Fruitloop *52* mutant	Non-toxic	++
+ Toxic control	--	pExTra02	Fruitloop *52*	Toxic	-
1		pExTra- Amelie 51	Amelie 51 replicate 1	Non-toxic	++
2		pExTra- Amelie 51	Amelie 51 replicate 2	Non-toxic	++
3		pExTra- Amelie 51	Amelie 51 replicate 3	Non-toxic	++

*Key: NG (no growth) - (no pink color) +(faint pink color) ++(obvious pink color) +++ (dark pink color)

Gene *52*; Score *0*

Images taken after 5 days at 37 ºC

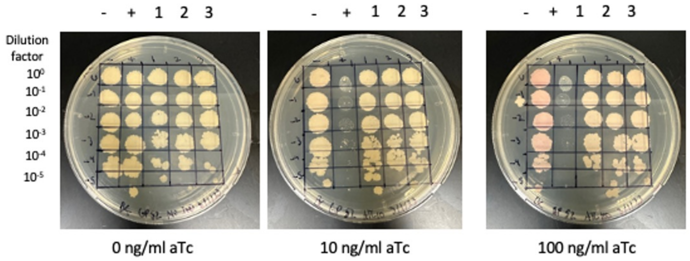

Lane	Gene ID	Plasmid name	Gene name	Toxic/Non-toxic	Colony color on 100 ng/ml aTc plate*
- Non-toxic control	--	pExTra03	Fruitloop *52* mutant	Non-toxic	+
+ Toxic control	--	pExTra02	Fruitloop *52*	Toxic	-
1	--	pExTra_52	Amelie *52* replicate 1	Non-toxic	+
2	--	pExTra_52	Amelie *52* replicate 2	Non-toxic	+
3	--	pExTra_52	Amelie *52* replicate 3	Non-toxic	+

*Key: NG (no growth) - (no pink color) +(faint pink color) ++(obvious pink color) +++ (dark pink color)

Gene *53*; Score *0*

Images taken after 5 days at 37 ºC

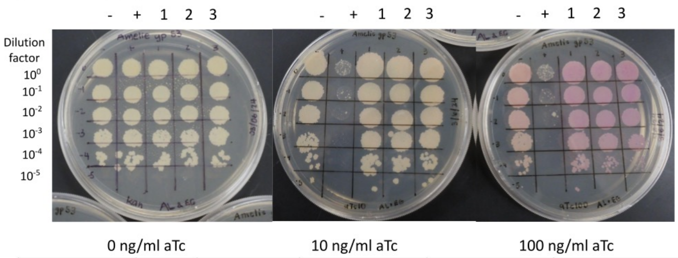

Lane	Gene ID	Plasmid name	Gene name	Toxic/Non-toxic	Colony color on 100 ng/ml aTc plate*
+ Toxic control	--	pExTra02	Fruitloop *52*	Toxic	-
- Non-toxic control	--	pExTra03	Fruitloop *52* mutant	Non-toxic	+
1		pExTra-Amelie53	Amelie *53* replicate 1	Non-toxic	++
2		pExTra-Amelie53	Amelie *53* replicate 2	Non-toxic	++
3		pExTra-Amelie53	Amelie *53* replicate 3	Non-toxic	++

*Key: NG (no growth) - (no pink color) +(faint pink color) ++(obvious pink color) +++ (dark pink color)

Gene *54*; Score *0*

Images taken after 5 days at 37 ºC

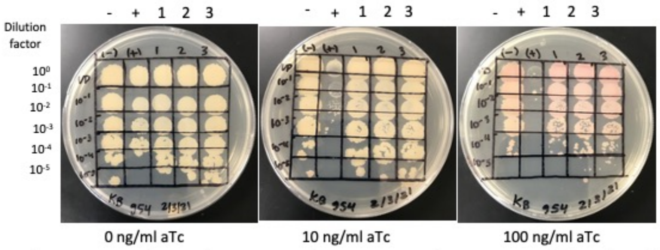

Lane	Gene ID	Plasmid name	Gene name	Toxic/Non-toxic	Colony color on 100 ng/ml aTc plate*
- Non-toxic control	--	pExTra03	Fruitloop *52* mutant	Non-toxic	++
+ Toxic control	--	pExTra02	Fruitloop *52*	Toxic	-
1	--	pExTra_54	Amelie 54 replicate 1	Non-toxic	+
2	--	pExTra_54	Amelie 54 replicate 2	Non-toxic	+
3	--	pExTra_54	Amelie 54 replicate 3	Non-toxic	+

*Key: NG (no growth) - (no pink color) +(faint pink color) ++(obvious pink color) +++ (dark pink color)

Gene *55*; Score *1*

Images taken after 4 days at 37 ºC

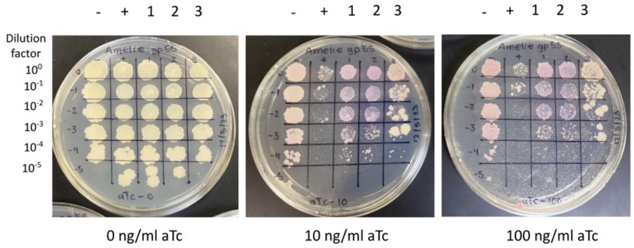

Lane	Gene ID	Plasmid name	Gene name	Toxic/Non-toxic	Colony color on 100 ng/ml aTc plate*
- Non-toxic control	--	pExTra03	Fruitloop *52* mutant	Non-toxic	++
+ Toxic control	--	pExTra02	Fruitloop *52*	Toxic	-
1		pExTra-Amelie55	Amelie *55* replicate 1	Toxic	+++
2		pExTra-Amelie55	Amelie *55* replicate 2	Toxic	+++
3		pExTra-Amelie55	Amelie *55* replicate 3	Toxic	+++

*Key: NG (no growth) - (no pink color) +(faint pink color) ++(obvious pink color) +++ (dark pink color)

Gene *56*; Score *1*

Images taken after 5 days at 37 ºC

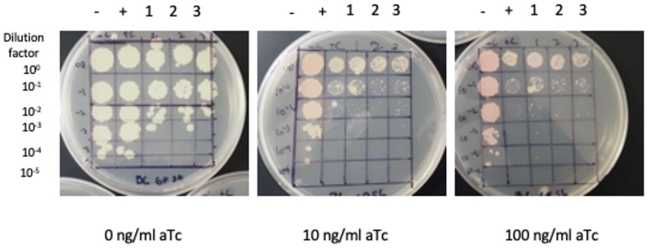

Lane	Gene ID	Plasmid name	Gene name	Toxic/Non-toxic	Colony color on 100 ng/ml aTc plate*
- Non-toxic control	--	pExTra03	Fruitloop *52* mutant	Non-toxic	++
+ Toxic control	--	pExTra02	Fruitloop *52*	Toxic	-
1	--	pExTra_56	Amelie *56* replicate 1	Toxic	+
2	--	pExTra_56	Amelie *56* replicate 2	Toxic	+
3	--	pExTra_56	Amelie *56* replicate 3	Toxic	+

*Key: NG (no growth) - (no pink color) +(faint pink color) ++(obvious pink color) +++ (dark pink color)

Gene *57*; Score *3*

Images taken after 5 days at 37 ºC

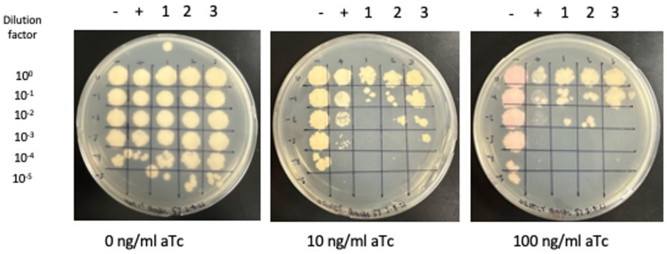

Lane	Gene ID	Plasmid name	Gene name	Toxic/Non-toxic	Colony color on 100 ng/ml aTc plate*
- Non-toxic control	--	pExTra03	Fruitloop *52* mutant	Non-toxic	+
+ Toxic control	--	pExTra02	Fruitloop *52*	Toxic	-
1		pExtra_57	Amelie 57 replicate 1	Toxic	+
2		pExtra_57	Amelie 57 replicate 2	Toxic	+
3		pExtra_57	Amelie 57 replicate 3	Toxic	+

*Key: NG (no growth) - (no pink color) +(faint pink color) ++(obvious pink color) +++ (dark pink color)

Gene 58; Score 1

Images taken after 5 days at 37 ºC

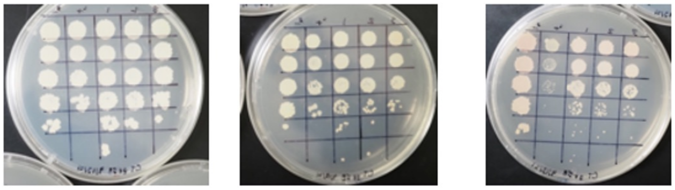

0 ng/ml aTc   10 ng/ml aTc   100 ng/ml aTc

Lane	Gene ID	Plasmid name	Gene name	Toxic/Non-toxic	Colony color on 100 ng/ml aTc plate*
- Non-toxic control	--	pExTra03	Fruitloop 52 mutant	Non-toxic	+
+ Toxic control	--	pExTra02	Fruitloop 52	Toxic	-
1	--	pExTra_58	Amelie 58 replicate 1	Toxic	+
2	--	pExTra_58	Amelie 58 replicate 2	Toxic	+
3	--	pExTra_58	Amelie 58 replicate 3	Toxic	+

*Key: NG (no growth) - (no pink color) +(faint pink color) ++(obvious pink color) +++ (dark pink color)

Gene 59; Score 0

Images taken after 5 days at 37°C

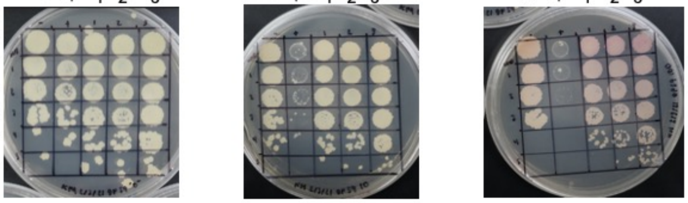

0 ng/ml aTc   10 ng/ml aTc   100 ng/ml aTc

Lane	Gene ID	Plasmid Name	Gene Name	Toxic/Non-Toxic	Colony Color on 100 ng/mL aTc Plate
(-) Non-Toxic Control	-----	pExTra_03	Fruitloop 52 Mutant	Non-Toxic	+
(+) Toxic Control	-----	pExTra_02	Fruitloop 52	Toxic	-
1	-----	pExTra_59	Amelie 59 Replicate 1	Non-Toxic	+
2	-----	pExTra_59	Amelie 59 Replicate 2	Non-Toxic	+
3	-----	pExTra_59	Amelie 59 Replicate 3	Non-Toxic	++

*Key: NG (No Growth) - (no pink color) + (faint pink color) ++ (obvious pink color) +++ (dark pink color)

Gene 60; Score 0

Images taken after 5 days at 37 ºC

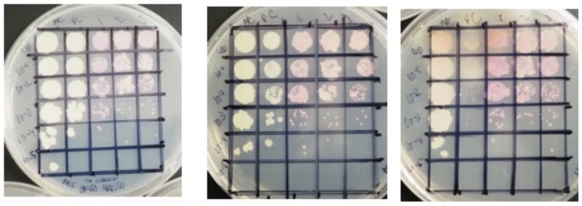

0 ng/ml aTc   10 ng/ml aTc   100 ng/ml aTc

Lane	Gene ID	Plasmid name	Gene name	Toxic/Non-toxic	Colony color on 100 ng/ml aTc plate*
- Non-toxic control	--	pExTra03	Fruitloop 52 mutant	Non-toxic	+
+ Toxic control	--	pExTra02	Fruitloop 52	Toxic	-
1	--	pExTra_60	Amelie 60 replicate 1	Non-toxic	++
2	--	pExTra_60	Amelie 60 replicate 2	Non-toxic	++
3	--	pExTra_60	Amelie 60 replicate 3	Non-toxic	++

*Key: NG (no growth) - (no pink color) +(faint pink color) ++(obvious pink color) +++ (dark pink color)

Gene 61; Score 0

Images taken after 5 days at 37 ºC

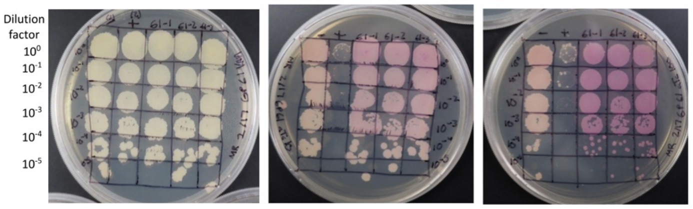

0 ng/ml aTc   10 ng/ml aTc   100 ng/ml aTc

Lane	Gene ID	Plasmid name	Gene name	Toxic/Non-toxic	Colony color on 100 ng/ml aTc plate*
- Non-toxic control	--	pExTra03	Fruitloop 52 mutant	Non-toxic	+
+ Toxic control	--	pExTra02	Fruitloop 52	Toxic	-
1		pExtra-Amelie_61	Amelie 61 replicate 1	Non-toxic	+++
2		pExtra-Amelie_61	Amelie 61 replicate 2	Non-toxic	+++
3		pExtra-Amelie_61	Amelie 61 replicate 3	Non-toxic	+++

*Key: NG (no growth) - (no pink color) +(faint pink color) ++(obvious pink color) +++ (dark pink color)

Gene 62; Score 2

Images taken after 5 days at 37 ºC

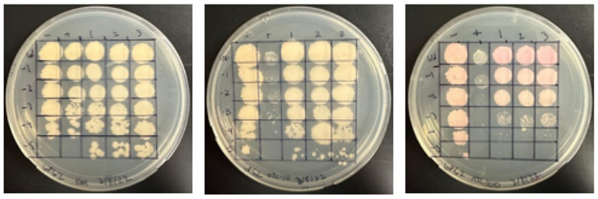

0 ng/ml aTc   10 ng/ml aTc   100 ng/ml aTc

Lane	Gene ID	Plasmid name	Gene name	Toxic/Non-toxic	Colony color on 100 ng/ml aTc plate*
- Non-toxic control	--	pExTra03	Fruitloop 52 mutant	Non-toxic	+
+ Toxic control	--	pExTra02	Fruitloop 52	Toxic	-
1		pExtra_62	Amelie 62 replicate 1	Toxic	++
2		pExtra_62	Amelie 62 replicate 2	Toxic	++
3		pExtra_62	Amelie 62 replicate 3	Toxic	++

*Key: NG (no growth) - (no pink color) +(faint pink color) ++(obvious pink color) +++ (dark pink color)

Gene 63; Score 1

Images taken after 4 days at 37 ºC

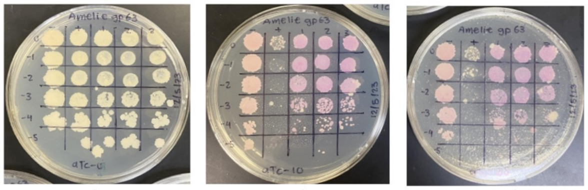

0 ng/ml aTc   10 ng/ml aTc   100 ng/ml aTc

Lane	Gene ID	Plasmid name	Gene name	Toxic/Non-toxic	Colony color on 100 ng/ml aTc plate*
- Non-toxic control	--	pExTra03	Fruitloop 52 mutant	Non-toxic	++
+ Toxic control	--	pExTra02	Fruitloop 52	Toxic	-
1		pExTra-Amelie63	Amelie 63 replicate 1	Toxic	+++
2		pExTra-Amelie63	Amelie 63 replicate 2	Toxic	+++
3		pExTra-Amelie63	Amelie 63 replicate 3	Toxic	+++

*Key: NG (no growth) - (no pink color) +(faint pink color) ++(obvious pink color) +++ (dark pink color)

Gene 64; Score 0

Images taken after 5 days at 37 ºC

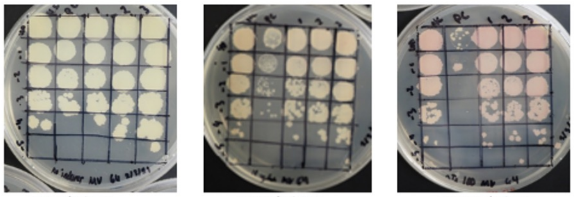

0 ng/ml aTc   10 ng/ml aTc   100 ng/ml aTc

Lane	Gene ID	Plasmid name	Gene name	Toxic/Non-toxic	Colony color on 100 ng/ml aTc plate*
- Non-toxic control	--	pExTra03	Fruitloop 52 mutant	Non-toxic	++
+ Toxic control	--	pExTra02	Fruitloop 52	Toxic	-
1	--	pExTra_64	Amelie 64 replicate 1	Non-toxic	++
2	--	pExTra_64	Amelie 64 replicate 2	Non-toxic	++
3	--	pExTra_64	Amelie 64 replicate 3	Non-toxic	++

*Key: NG (no growth) - (no pink color) +(faint pink color) ++(obvious pink color) +++ (dark pink color)

Gene 65; Score 1

Images taken after 4 days at 37 ºC

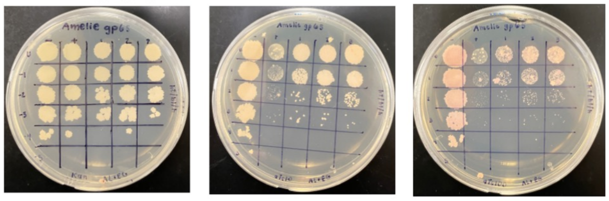

0 ng/ml aTc   10 ng/ml aTc   100 ng/ml aTc

Lane	Gene ID	Plasmid name	Gene name	Toxic/Non-toxic	Colony color on 100 ng/ml aTc plate*
+ Toxic control	--	pExTra02	Fruitloop 52	Toxic	+
- Non-toxic control	--	pExTra03	Fruitloop 52 mutant	Non-toxic	-
1	131436	pExTra-Amelie65	Amelie 65 replicate 1	Toxic	+
2	131436	pExTra-Amelie65	Amelie 65 replicate 2	Toxic	+
3	131436	pExTra-Amelie65	Amelie 65 replicate 3	Toxic	+

*Key: NG (no growth) - (no pink color) +(faint pink color) ++(obvious pink color) +++ (dark pink color)

Images taken after 5 days at 37 ºC

Gene *66*; Score *0*

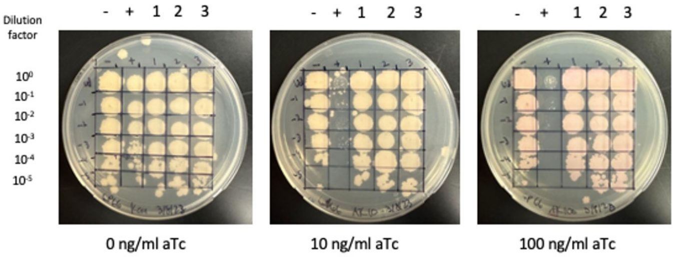

Lane	Gene ID	Plasmid name	Gene name	Toxic/Non-toxic	Colony color on 100 ng/ml aTc plate*
- Non-toxic control	--	pExTra03	Fruitloop *52* mutant	Non-toxic	+
+ Toxic control	--	pExTra02	Fruitloop *52*	Toxic	-
1		pExtra_66	Amelie 66 replicate 1	Non-toxic	++
2		pExtra_66	Amelie 66 replicate 2	Non-toxic	++
3		pExtra_66	Amelie 66 replicate 3	Non-toxic	++

*Key: NG (no growth) - (no pink color) +(faint pink color) ++(obvious pink color) +++ (dark pink color)

Images taken after 5 days at 37 ºC

Gene *67*; Score *0*

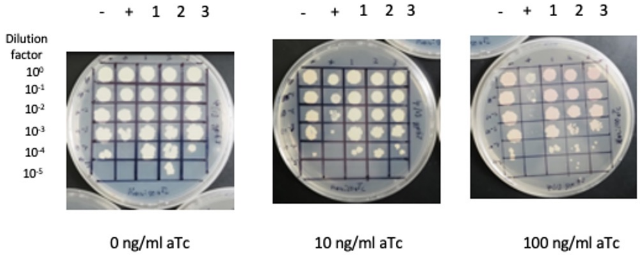

Lane	Gene ID	Plasmid name	Gene name	Toxic/Non-toxic	Colony color on 100 ng/ml aTc plate*
- Non-toxic control	--	pExTra03	Fruitloop *52* mutant	Non-toxic	+
+ Toxic control	--	pExTra02	Fruitloop *52*	Toxic	-
1	--	pExTra_67	Amelie *67* replicate 1	Non-toxic	+
2	--	pExTra_67	Amelie *67* replicate 2	Non-toxic	+
3	--	pExTra_67	Amelie *67* replicate 3	Non-toxic	+

*Key: NG (no growth) - (no pink color) +(faint pink color) ++(obvious pink color) +++ (dark pink color)

Images taken after 5 days at 37 ºC

Gene *68*; Score *0*

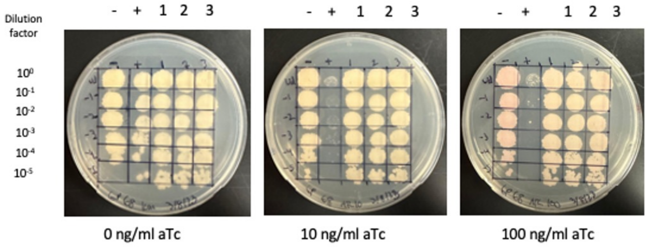

Lane	Gene ID	Plasmid name	Gene name	Toxic/Non-toxic	Colony color on 100 ng/ml aTc plate*
- Non-toxic control	--	pExTra03	Fruitloop *52* mutant	Non-toxic	+
+ Toxic control	--	pExTra02	Fruitloop *52*	Toxic	-
1		pExtra_68	Amelie 68 replicate 1	Non-toxic	+
2		pExtra_68	Amelie 68 replicate 2	Non-toxic	+
3		pExtra_68	Amelie 68 replicate 3	Non-toxic	+

*Key: NG (no growth) - (no pink color) +(faint pink color) ++(obvious pink color) +++ (dark pink color)

Images taken after 5 days at 37 ºC

Gene *69*; Score *0*

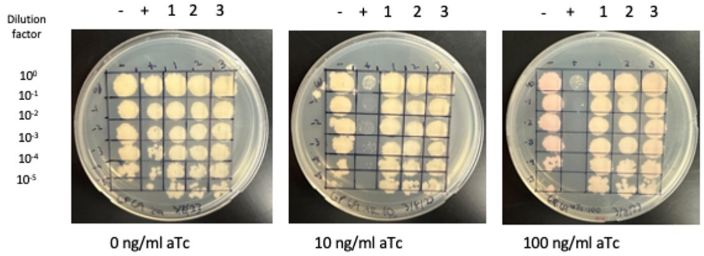

Lane	Gene ID	Plasmid name	Gene name	Toxic/Non-toxic	Colony color on 100 ng/ml aTc plate*
- Non-toxic control	--	pExTra03	Fruitloop *52* mutant	Non-toxic	+
+ Toxic control	--	pExTra02	Fruitloop *52*	Toxic	-
1		pExtra_69	Amelie 69 replicate 1	Non-toxic	+
2		pExtra_69	Amelie 69 replicate 2	Non-toxic	+
3		pExtra_69	Amelie 69 replicate 3	Non-toxic	+

*Key: NG (no growth) - (no pink color) +(faint pink color) ++(obvious pink color) +++ (dark pink color)

Images taken after 5 days at 37 ºC

Gene *70*; Score *0*

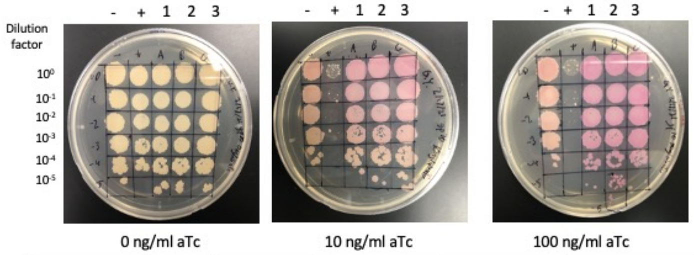

Lane	Gene ID	Plasmid name	Gene name	Toxic/Non-toxic	Colony color on 100 ng/ml aTc plate*
- Non-toxic control	--	pExTra03	Fruitloop *52* mutant	Non-toxic	++
+ Toxic control	--	pExTra02	Fruitloop *52*	Toxic	-
1	--	pExtra_70	Amelie 70 replicate 1	Non-toxic	+++
2	--	pExtra_70	Amelie 70 replicate 2	Non-toxic	+++
3	--	pExtra_70	Amelie 70 replicate 3	Non-toxic	+++

*Key: NG (no growth) - (no pink color) +(faint pink color) ++(obvious pink color) +++ (dark pink color)

Images taken after 5 days at 37 ºC

Gene *71*; Score *0*

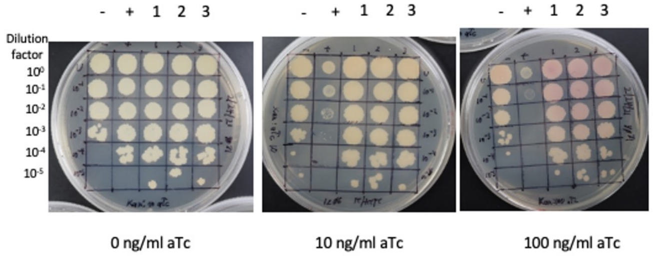

Lane	Gene ID	Plasmid name	Gene name	Toxic/Non-toxic	Colony color on 100 ng/ml aTc plate*
- Non-toxic control	--	pExTra03	Fruitloop *52* mutant	Non-toxic	+
+ Toxic control	--	pExTra02	Fruitloop *52*	Toxic	-
1	--	pExTra_71	Amelie *71* replicate 1	Non-toxic	+
2	--	pExTra_71	Amelie *71* replicate 2	Non-toxic	+
3	--	pExTra_71	Amelie *71* replicate 3	Non-toxic	+

*Key: NG (no growth) - (no pink color) +(faint pink color) ++(obvious pink color) +++ (dark pink color)

Images taken after 5 days at 37 ºC

Gene *72*; Score *0*

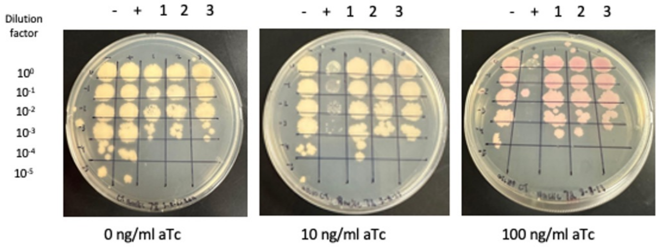

Lane	Gene ID	Plasmid name	Gene name	Toxic/Non-toxic	Colony color on 100 ng/ml aTc plate*
- Non-toxic control	--	pExTra03	Fruitloop *52* mutant	Non-toxic	+
+ Toxic control	--	pExTra02	Fruitloop *52*	Toxic	-
1		pExtra_72	Amelie 72 replicate 1	Non-toxic	++
2		pExtra_72	Amelie 72 replicate 2	Non-toxic	++
3		pExtra_72	Amelie 72 replicate 3	Non-toxic	++

*Key: NG (no growth) - (no pink color) +(faint pink color) ++(obvious pink color) +++ (dark pink color)

Images taken after 5 days at 37 ºC

Gene *73*; Score *3*

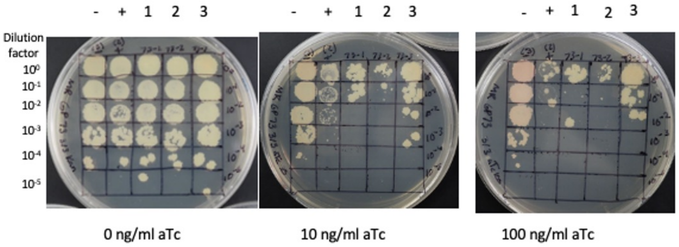

Lane	Gene ID	Plasmid name	Gene name	Toxic/Non-toxic	Colony color on 100 ng/ml aTc plate*
- Non-toxic control	--	pExTra03	Fruitloop *52* mutant	Non-toxic	+
+ Toxic control	--	pExTra02	Fruitloop *52*	Toxic	-
1		pExtra_73	Amelie 73 replicate 1	Toxic	-
2		pExtra_73	Amelie 73 replicate 2	Toxic	-
3		pExtra_73	Amelie 73 replicate 3	Toxic	-

*Key: NG (no growth) - (no pink color) +(faint pink color) ++(obvious pink color) +++ (dark pink color)

Gene *74*; Score *1*

Images taken after 5 days at 37 ºC

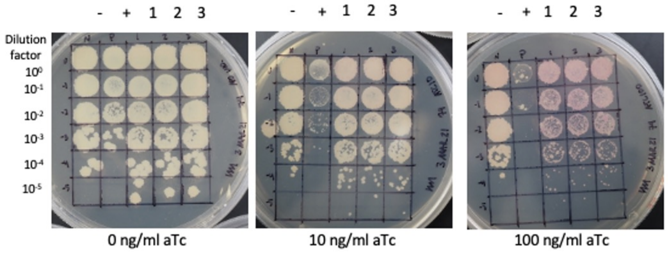

Lane	Gene ID	Plasmid name	Gene name	Toxic/Non-toxic	Colony color on 100 ng/ml aTc plate*
- Non-toxic control	--	pExTra03	Fruitloop *52* mutant	Non-toxic	++
+ Toxic control	--	pExTra02	Fruitloop *52*	Toxic	+
1	--	pExTra_74	Amelie *74*replicate 1	Toxic	+
2	--	pExTra_74	Amelie *74* replicate 2	Toxic	+
3	--	pExTra_74	Amelie *74* replicate 3	Toxic	+

*Key: NG (no growth) - (no pink color) +(faint pink color) ++(obvious pink color) +++ (dark pink color)

Gene *76*; Score *0*

Images taken after 5 days at 37 ºC

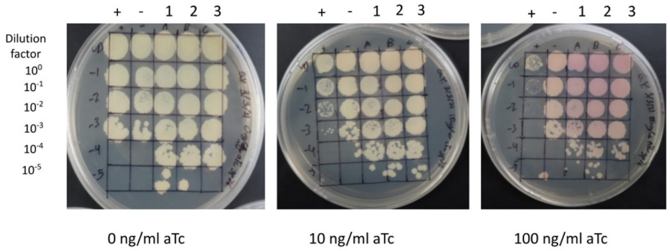

Lane	Gene ID	Plasmid name	Gene name	Toxic/Non-toxic	Colony color on 100 ng/ml aTc plate*
- Non-toxic control	--	pExTra03	Fruitloop *52* mutant	Non-toxic	+
+ Toxic control	--	pExTra02	Fruitloop *52*	Toxic	-
1	--	pExTra_76	Amelie 76 replicate 1	Non-toxic	++
2	--	pExTra_76	Amelie 76 replicate 2	Non-toxic	++
3	--	pExTra_76	Amelie 76 replicate 3	Non-toxic	++

*Key: NG (no growth) - (no pink color) +(faint pink color) ++(obvious pink color) +++ (dark pink color)

Gene *77*; Score *0*

Images taken after 5 days at 37 ºC

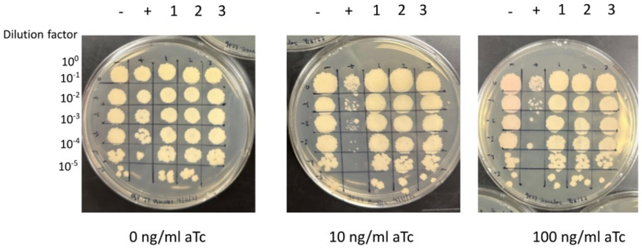

Lane	Gene ID	Plasmid name	Gene name	Toxic/Non-toxic	Colony color on 100 ng/ml aTc plate*
- Non-toxic control	--	pExTra03	Fruitloop *52* mutant	Non-toxic	+
+ Toxic control	--	pExTra02	Fruitloop *52*	Toxic	-
1	--	pExTra_77	Amelie *77* replicate 1	Non-toxic	+
2	--	pExTra_77	Amelie *77* replicate 2	Non-toxic	+
3	--	pExTra_77	Amelie *77* replicate 3	Non-toxic	+

*Key: NG (no growth) - (no pink color) +(faint pink color) ++(obvious pink color) +++ (dark pink color)

Supplemental Table 1: DNA oligos used in this study

Oligo Name	Oligo Sequence (5’ to 3’)
oAmelie1 _F	atgcggaggaatcacttccatATGTCGCTCGCGCCC
oAmelie1 _R	tgcaggatccgactcgagtgtcgacTCAGCCGGAAGGTGCGC
oAmelie2 _F	atgcggaggaatcacttccatATGCGCGCACCCGCTAC
oAmelie2 _R	tgcaggatccgactcgagtgtcgacTCACTCATCGCGGCCAC
oAmelie3 _F	atgcggaggaatcacttccatATGGCCGCGATGAGTGAC
oAmelie3 _R	tgcaggatccgactcgagtgtcgacTCAGAGGTCCGAGCCATC
oAmelie4 _F	atgcggaggaatcacttccatATGGCTCGGACCTCTGAG
oAmelie4 _R	tgcaggatccgactcgagtgtcgacTCACACGAACATTGCGCC
oAmelie5 _F	atgcggaggaatcacttccatATGATCCCGCAGCCTTTCG
oAmelie5 _R	tgcaggatccgactcgagtgtcgacTCACTGAGCCGAACCAGC
oAmelie6 _F	atgcggaggaatcacttccatATGACGGAACCCAAGGC
oAmelie6 _R	tgcaggatccgactcgagtgtcgacTCACTGCAGGAAGTCCTC
oAmelie7 _F	atgcggaggaatcacttccatATGAACCGCGACGAGCTG
oAmelie7 _R	tgcaggatccgactcgagtgtcgacTCACTCAGCCTGTGCCTG
oAmelie8 _F	atgcggaggaatcacttccatATGGCTGATAACGCGAGCG
oAmelie8 _R	tgcaggatccgactcgagtgtcgacTCAGTCGCCGGAACGCAG
oAmelie9 _F	atgcggaggaatcacttccatATGGCTGACATTTCACGCTCC
oAmelie9 _R	tgcaggatccgactcgagtgtcgacTCAGTCCCCTGCCGGGAG
oAmelie10 _F	atgcggaggaatcacttccatATGCTGGCGACGCTGGAC
oAmelie10 _R	tgcaggatccgactcgagtgtcgacTCAGTACCTCTCGCTGCCC
oAmelie11 _F	atgcggaggaatcacttccatATGTTCCCGACGCCCCAC
oAmelie11 _R	tgcaggatccgactcgagtgtcgacTCAAGCGGTCGGTATGGC
oAmelie12 _F	atgcggaggaatcacttccatATGCCATACCGACCGCTTG
oAmelie12 _R	tgcaggatccgactcgagtgtcgacTCACCGTGGCCCCAACTC
oAmelie13 _F	atgcggaggaatcacttccatATGAGCGTGCTCGTTCCGC
oAmelie13 _R	tgcaggatccgactcgagtgtcgacTCAGCTGCGGCGCTCGG
oAmelie14 _F	atgcggaggaatcacttccatATGGCAGACGAGCCTACTTC
oAmelie14 _R	tgcaggatccgactcgagtgtcgacTCAGACGCCGATCTTCTGAC

oAmelie15 _F	atgcggaggaatcacttccatATGACTGAGACCAACGTCGACAGC
oAmelie15 _R	tgcaggatccgactcgagtgtcgacTCACCTGCCGCGCCGCC
oAmelie16 _F	atgcggaggaatcacttccatATGACTGAGACCAACGTCGAC
oAmelie16 _R	tgcaggatccgactcgagtgtcgacTCACGCCCCCTTGCTCTTG
oAmelie17 _F	atgcggaggaatcacttccatATGAGCGCTACGTACTACCTC
oAmelie17 _R	tgcaggatccgactcgagtgtcgacTCACCTGTATCGAGCGCGAG
oAmelie18 _F	atgcggaggaatcacttccatATGGACAAGAAGTACACGGGC
oAmelie18 _R	tgcaggatccgactcgagtgtcgacTCACGACCACGCCATCCG
oAmelie19 _F	atgcggaggaatcacttccatATGTACGTGAAAGATGGCCGC
oAmelie19 _R	tgcaggatccgactcgagtgtcgacTCAGAACATGTCTCCTGATCCG
oAmelie20 _F	atgcggaggaatcacttccatATGGAAATGCCCACACTGCCAC
oAmelie20 _R	tgcaggatccgactcgagtgtcgacTCACTCGTCGAAAGTGATGGAC
oAmelie21 _F	atgcggaggaatcacttccatATGGCCGAGCTTGCGC
oAmelie21 _R	tgcaggatccgactcgagtgtcgacTCACTCCCCTTGCGGCAC
oAmelie22 _F	atgcggaggaatcacttccatATGCCGTACACCAAGAGTTACC
oAmelie22 _R	tgcaggatccgactcgagtgtcgacTCACTTACCTCCTGCCTTGC
oAmelie23 _F	atgcggaggaatcacttccatATGCCTCCCGTCTACGACC
oAmelie23 _R	tgcaggatccgactcgagtgtcgacTCACGTCGTCGCCGTGAC
oAmelie24 _F	atgcggaggaatcacttccatATGGCGTGGTCAACAAACCC
oAmelie24 _R	tgcaggatccgactcgagtgtcgacTCACTGGTAGAACCTCAGCCAG
oAmelie25 _F	atgcggaggaatcacttccatATGGCAGGAGCGGCTG
oAmelie25 _R	tgcaggatccgactcgagtgtcgacTCAGTCCGTCAGGTCGTAGATG
oAmelie26 _F	atgcggaggaatcacttccatATGATTATCTACCGCGCTAACGTC
oAmelie26 _R	tgcaggatccgactcgagtgtcgacTCACGCGCTCACCCC
oAmelie27 _F	atgcggaggaatcacttccatATGAGCAAGCCTGTTCTGCTC
oAmelie27 _R	tgcaggatccgactcgagtgtcgacTCACGCCGCCATCGC
oAmelie28 _F	atgcggaggaatcacttccatATGGCGAAATCGGCCGC
oAmelie28 _R	tgcaggatccgactcgagtgtcgacTCACCGCTCGACCGCC
oAmelie29 _F	atgcggaggaatcacttccatATGAGCGCCGACACGTTG
oAmelie29 _R	tgcaggatccgactcgagtgtcgacTCATTCGATCGCCCCGTCG

oAmelie30 _F	atgcggaggaatcacttccatATGAGCCTCGCAGAACG
oAmelie30 _R	tgcaggatccgactcgagtgtcgacTCAGACAGCGACACGGG
oAmelie31 _F	atgcggaggaatcacttccatATGTCGCTGTCTGACCGAC
oAmelie31 _R	tgcaggatccgactcgagtgtcgacTCACACGACGCTGAGGC
oAmelie32 _F	atgcggaggaatcacttccatATGAACGCTCACACAATGACAG
oAmelie32 _R	tgcaggatccgactcgagtgtcgacTCACCGATCCTTCTTGCCC
oAmelie33 _F	atgcggaggaatcacttccatATGGCCGGGAGTGATGTTC
oAmelie33 _R	tgcaggatccgactcgagtgtcgacTCAGTGATCGCAGACGTCG
oAmelie34 _F	atgcggaggaatcacttccatATGAGCGAGTACACCAAGGAC
oAmelie34 _R	tgcaggatccgactcgagtgtcgacTCACTTGTTCTTTGGCAGCTTG
oAmelie35 _F	atgcggaggaatcacttccatATGATCGAGCACTTTTACCTCGG
oAmelie35 _R	tgcaggatccgactcgagtgtcgacTCAGGCGACGTCGAACAAC
oAmelie36 _F	atgcggaggaatcacttccatATGGCATCGCTTCGCACC
oAmelie36 _R	tgcaggatccgactcgagtgtcgacTCAGTTCCGGTCGAGTTGG
oAmelie37 _F	atgcggaggaatcacttccatATGCTCGACGATCTCAATAGAATC
oAmelie37 _R	tgcaggatccgactcgagtgtcgacTCAGTGGGTTGACTGCAGG
oAmelie38 _F	atgcggaggaatcacttccatATGCCAGACCATGACGACG
oAmelie38 _R	tgcaggatccgactcgagtgtcgacTCACCGCAGAATGGCGTCC
oAmelie39 _F	atgcggaggaatcacttccatATGCAGAACTTTAGACACGAACTGC
oAmelie39 _R	tgcaggatccgactcgagtgtcgacTCATGCGGCGGCCCG
oAmelie40 _F	atgcggaggaatcacttccatATGAGTTCTCCCGCAACCG
oAmelie40 _R	tgcaggatccgactcgagtgtcgacTCAACTGCATGAGCGGCTC
oAmelie41 _F	atgcggaggaatcacttccatATGCAGTTAGCCCGCCAC
oAmelie41 _R	tgcaggatccgactcgagtgtcgacTCATTTCTCGGCTCCCCC
oAmelie42 _F	atgcggaggaatcacttccatATGCCGAGCAGAATCTTAGCG
oAmelie42 _R	tgcaggatccgactcgagtgtcgacTCATCGAGCGACCGCC
oAmelie43 _F	atgcggaggaatcacttccatATGACCCCGGCGCCG
oAmelie43 _R	tgcaggatccgactcgagtgtcgacTCACCAAGGCAGCACCC
oAmelie44 _F	atgcggaggaatcacttccatATGGTGACCCTGACTCACG
oAmelie44 _R	tgcaggatccgactcgagtgtcgacTCACAGCATCACCGTCCG

oAmelie45 _F	atgcggaggaatcacttccatATGACGGTTCGGCGCATC
oAmelie45 _R	tgcaggatccgactcgagtgtcgacTCACGCTGCGCCCTCG
oAmelie46 _F	atgcggaggaatcacttccatATGACCGGCGGCCCATTC
oAmelie46 _R	tgcaggatccgactcgagtgtcgacTCACGCGACCACCTCC
oAmelie47 _F	atgcggaggaatcacttccatATGACCGACCGGCCGC
oAmelie47 _R	tgcaggatccgactcgagtgtcgacTCATTCAGTTGTGCTCAGCGC
oAmelie48 _F	atgcggaggaatcacttccatATGGCTGACGTTTCGGACC
oAmelie48 _R	tgcaggatccgactcgagtgtcgacTCATCCCTCTTCTTCGACCAG
oAmelie49 _F	atgcggaggaatcacttccatATGAGCGCAAGGAATCTGATCG
oAmelie49 _R	tgcaggatccgactcgagtgtcgacTCACAGGTGCGCCCC
oAmelie50 _F	atgcggaggaatcacttccatATGAGCGCCAATGCGAAGTTC
oAmelie50 _R	tgcaggatccgactcgagtgtcgacTCACGCGACGTCTCCC
oAmelie51 _F	atgcggaggaatcacttccatATGAGGCGCCCGGCGC
oAmelie51 _R	tgcaggatccgactcgagtgtcgacTCAGGCCGCGGATTCGC
oAmelie52 _F	atgcggaggaatcacttccatATGCGCAAGGTAATTGCCGTG
oAmelie52 _R	tgcaggatccgactcgagtgtcgacTCACAGCAAGATGAAGCTGCC
oAmelie53 _F	atgcggaggaatcacttccatATGAGCCGCCATAACTGCAG
oAmelie53 _R	tgcaggatccgactcgagtgtcgacTCACGTCCCGTCGTAGAAAC
oAmelie54 _F	atgcggaggaatcacttccatATGAGCAATGACTCGTACGACTTC
oAmelie54 _R	tgcaggatccgactcgagtgtcgacTCACTGCTTCGCTGCCAG
oAmelie55 _F	atgcggaggaatcacttccatATGATCACGATTTACACGACCG
oAmelie55 _R	tgcaggatccgactcgagtgtcgacTCACGCTGCCTGCCG
oAmelie56 _F	atgcggaggaatcacttccatATGAACACGACGGCCCTG
oAmelie56 _R	tgcaggatccgactcgagtgtcgacTCAAAGGGTTGGGCGGC
oAmelie57 _F	atgcggaggaatcacttccatATGAACGGACTTTCTGACCTGC
oAmelie57 _R	tgcaggatccgactcgagtgtcgacTCACCGCCGGAAACCG
oAmelie58 _F	atgcggaggaatcacttccatATGCACCTCGACCACACAAC
oAmelie58 _R	tgcaggatccgactcgagtgtcgacTCAGGCATCCCCAGCC
oAmelie59 _F	atgcggaggaatcacttccatATGCCTGATCTGATCGAGTTG
oAmelie59 _R	tgcaggatccgactcgagtgtcgacTCACTTCGGCGACACCTC

oAmelie60 _F	atgcggaggaatcacttccatATGACTTTGCCGGTTTCTGTGG
oAmelie60 _R	tgcaggatccgactcgagtgtcgacTCATCCCATCACCTGACTAGG
oAmelie61 _F	atgcggaggaatcacttccatATGTACACAGAGGCATGGTTGTCG
oAmelie61 _R	tgcaggatccgactcgagtgtcgacTCACGCCAGCGCCGC
oAmelie62 _F	atgcggaggaatcacttccatATGAAGCGTATGAAGGCGTTTCG
oAmelie62 _R	tgcaggatccgactcgagtgtcgacTCAGGCGACGTTCGCCC
oAmelie63 _F	atgcggaggaatcacttccatATGAAGGTCAGCAAGGTTCG
oAmelie63 _R	tgcaggatccgactcgagtgtcgacTCAGGCGCCGATGCTG
oAmelie64 _F	atgcggaggaatcacttccatATGAACACTCTTCACCTCACGG
oAmelie64 _R	tgcaggatccgactcgagtgtcgacTCATCGGGCGACCTCGC
oAmelie65 _F	atgcggaggaatcacttccatATGAGCGCCGAGGCC
oAmelie65 _R	tgcaggatccgactcgagtgtcgacTCACGCAACCACCTCCG
oAmelie66 _F	atgcggaggaatcacttccatATGAGCGCCGCCGAGG
oAmelie66 _R	tgcaggatccgactcgagtgtcgacTCACACCCAGCCGGGCAC
oAmelie67 _F	atgcggaggaatcacttccatATGTCGCCGACGGTAATCAAC
oAmelie67 _R	tgcaggatccgactcgagtgtcgacTCACAGCCCCTTCACGTTG
oAmelie68 _F	atgcggaggaatcacttccatATGGCCGACTATTCGTTCGC
oAmelie68 _R	tgcaggatccgactcgagtgtcgacTCACCCATGCCGCAGCAC
oAmelie69 _F	atgcggaggaatcacttccatATGGGTAGTGCGCCGGTG
oAmelie69 _R	tgcaggatccgactcgagtgtcgacTCACTCATCAGCCACCACC
oAmelie70 _F	atgcggaggaatcacttccatATGAGTGATGTTGTTGATACGCG
oAmelie70 _R	tgcaggatccgactcgagtgtcgacTCAGCCAAACAGGCGCC
oAmelie71 _F	atgcggaggaatcacttccatATGTTCAAGATGATCGTTCAGCTTC
oAmelie71 _R	tgcaggatccgactcgagtgtcgacTCACGCGGCCGCCAG
oAmelie72 _F	atgcggaggaatcacttccatATGGACGCTCCAACGCATTTC
oAmelie72 _R	tgcaggatccgactcgagtgtcgacTCAGTCGGTGTACTTCACCTCG
oAmelie73 _F	atgcggaggaatcacttccatATGCCGAAACGTACCGAGG
oAmelie73 _R	tgcaggatccgactcgagtgtcgacTCAGCGCCACCAGCC
oAmelie74 _F	atgcggaggaatcacttccatATGATGGTTAAGGGTATCGAGTGG
oAmelie74 _R	tgcaggatccgactcgagtgtcgacTCAGCAGCCGTGCACCAG

oAmelie75 _F	atgcggaggaatcacttccatATGTCGACCACCTTCAAGTACC
oAmelie75 _R	tgcaggatccgactcgagtgtcgacTCAGTCCTCGGCCGC
oAmelie76 _F	atgcggaggaatcacttccatATGTCCGAGAACGCTCCC
oAmelie76 _R	tgcaggatccgactcgagtgtcgacTCACCAGTCGTACTGCTGGTAG
oAmelie77 _F	atgcggaggaatcacttccatATGACGGCGGGCCGC
oAmelie77 _R	tgcaggatccgactcgagtgtcgacTCAGGGTCGCCACGTCC
pExTra_F	GTCGACACTCGAGTCGGATCCTG
pExTra_R	ATGGAAGTGATTCCTCCGCATGC
pExTra_seqF	GTACCCGTGTGTACGACCAGC
pExTra_uniR	CCCTTCGAGACCATAGATCTGTTCC
oAmelie_6ia_seq	GGACACGATCACTGCAAGTGC
oAmelie_6ib_seq	GTTTCGCACGCTTGGGTG
oAmelie_17ia_seq	AGCGTTCGGGCTCGACG
oAmelie_17ib_seq	AGCGAGTCATTGACTTCATGGGG
oAmelie_17ic_seq	GTTCCTCATCGGCCTGCATC
oAmelie_17id_seq	TGGCGAACCGAGAACCGAAC
oAmelie_17ie_seq	CAGTGGTTGTGGAATGCGGC
oAmelie_23ia_seq	CCTGACCGCAGCCAACG
oAmelie_23ib_seq	CCATCCCGAAGCCCCAG
oAmelie_23ic_seq	GGACGTGCACCAGATCCTC
oAmelie_24ia_seq	GTCTGCTGCTCAACGGTCG
oAmelie_57ia_seq	CCGGGCAGCTACAACATGAAG
oAmelie_57ib_seq	CATGTACCGGCCGCTGAG
